# Functional and Adaptive Significance of Promoter Mutations That Affect Divergent Myocardial Expressions of *TRIM72* in Primates

**DOI:** 10.1093/molbev/msab083

**Published:** 2021-03-21

**Authors:** Yuanqing Feng, Hongzhan Xu, Jinghao Liu, Ning Xie, Lei Gao, Yanyun He, Yuan Yao, Fengxiang Lv, Yan Zhang, Jian Lu, Wei Zhang, Chuan-Yun Li, Xinli Hu, Ziheng Yang, Rui-Ping Xiao

**Affiliations:** 1 Institute of Molecular Medicine, College of Future Technology, Peking University, Beijing, China; 2 College of Animal Science and Technology, China Agricultural University, Beijing, China; 3 Peking-Tsinghua Center for Life Sciences, Beijing, China; 4 State Key Laboratory of Protein and Plant Gene Research, Beijing, China; 5 School of Life Sciences, Peking University, Beijing, China; 6 Department of Genetics, Evolution and Environment, University College London, London, United Kingdom; 7 State Key Laboratory of Biomembrane and Membrane Biotechnology, Beijing, China; 8 Beijing Key Laboratory of Cardiometabolic Molecular Medicine, Peking University, Beijing, China

**Keywords:** TRIM72, promoter mutations, primate evolution, heart metabolism

## Abstract

Cis-regulatory elements play important roles in tissue-specific gene expression and in the evolution of various phenotypes, and mutations in promoters and enhancers may be responsible for adaptations of species to environments. TRIM72 is a highly conserved protein that is involved in energy metabolism. Its expression in the heart varies considerably in primates, with high levels of expression in Old World monkeys and near absence in hominids. Here, we combine phylogenetic hypothesis testing and experimentation to demonstrate that mutations in promoter are responsible for the differences among primate species in the heart-specific expression of *TRIM72*. Maximum likelihood estimates of lineage-specific substitution rates under local-clock models show that relative to the evolutionary rate of introns, the rate of promoter was accelerated by 78% in the common ancestor of Old World monkeys, suggesting a role for positive selection in the evolution of the *TRIM72* promoter, possibly driven by selective pressure due to changes in cardiac physiology after species divergence. We demonstrate that mutations in the *TRIM72* promoter account for the differential myocardial *TRIM72* expression of the human and the rhesus macaque. Furthermore, changes in *TRIM72* expression alter the expression of genes involved in oxidative phosphorylation, which in turn affects mitochondrial respiration and cardiac energy capacity. On a broader timescale, phylogenetic regression analyses of data from 29 mammalian species show that mammals with high cardiac expression of *TRIM72* have high heart rate, suggesting that the expression changes of *TRIM72* may be related to differences in the heart physiology of those species.

## Introduction

The heart is a classic example of morphological evolution in vertebrates. The genetic mechanism underlying anatomical changes from single tubular to multi-chambered hearts in major chordate groups (fishes, amphibians, reptiles, birds, mammals) has been studied extensively (Moorman and Christoffels 2003; Olson 2006; Stephenson et al. 2017). In birds and mammals, the four-chambered heart separates oxygen-rich pulmonary and oxygen-poor systemic circuits; the increased heart rate and relative ventricle mass also help to increase blood flow and stroke volume, allowing for high metabolic rates and endothermy (Hillenius and Ruben 2004; Seymour et al. 2004; Hillman and Hedrick 2015a). Extensive studies have revealed that altered cardiac expressions of transcription factors (e.g., NKX2-5, TBX5) play important roles in the anatomical/morphological evolution of the vertebrate heart (Olson 2006; Koshiba-Takeuchi et al. 2009). Among mammals, there have been no obvious changes in the anatomical structure of the heart. However, mammals show a huge range of heart rate (10–1,000 beats per minute), which may necessitate considerable remodeling of energy metabolism in cardiomyocytes. Currently, little is known about the functional evolution of the heart in different mammalian species, and especially in primates. 

Changes in the cis-regulatory elements, especially promoters and enhancers, may cause spatiotemporal alterations in gene expression and contribute to phenotypic differences within and between species (Wray 2007; Necsulea and Kaessmann 2014; Boyd et al. 2015; Reilly et al. 2015; Reilly and Noonan 2016). Regulatory changes, which may be under tissue-specific natural selection, have long been hypothesized to play an important role in primate evolution (King and Wilson 1975; Haygood et al. 2007; Blekhman et al. 2008; Prabhakar et al. 2008; Shibata et al. 2012; Boyd et al. 2015). However, detecting adaptive changes in noncoding regulatory regions is challenging (Wray et al. 2003; Andolfatto 2005; Blekhman et al. 2008). Functionally important regulatory elements are often short genomic segments, so that statistical tests often have low power. A major difficulty is that a reference is often lacking in comparative analysis of noncoding regions, unlike protein-coding genes, in which synonymous substitutions act as a natural benchmark against which acceleration of nonsynonymous substitutions driven by positive selection may be detected (Yang 2019). Higher rates of evolution in noncoding regulatory regions may be due to local mutation rate variation and relaxed selective constraint, as well as positive selection driving the fixation of advantageous mutations, and those factors may be hard to distinguish. It has been suggested that neighboring introns or silent sites in coding genes may be used as a reference for inference of selection-driven rate acceleration in noncoding regulatory elements (Andolfatto 2005).

TRIM72 (also known as MG53) belongs to the tripartite motif-containing (TRIM) family of proteins, and is predominantly expressed in the skeletal muscle in mammals (Cai et al. 2009; Song et al. 2013). Previously, Song et al. (2013) showed that TRIM72 acts as an E3 ligase targeting insulin receptor and insulin receptor substrate 1 for degradation, with abnormal *TRIM72* expression causing insulin resistance and metabolic disorders. In rodents, TRIM72 not only affects pre- and post-conditioning against myocardial ischemia reperfusion injury, but also contributes to the development of diabetic cardiomyopathy (Cao et al. 2010; Zhang et al. 2011; Liu et al. 2015). TRIM72 is also shown to regulate lipid metabolism by modulating the expression of PPARα in murine hearts (Liu et al. 2015). However, the expression level of *TRIM72* in the heart varies dramatically among different mammals, with high expression in mice and rhesus monkeys, and low expression in pigs, sheep, and human (Song et al. 2013; Liu et al. 2015; Lemckert et al. 2016). It is then interesting to ask whether such tissue-specific changes in expression among species have been driven by natural selection fixing advantageous mutations and what functional changes underlie such dramatic changes in expression. As TRIM72 is involved in energy metabolism, and the heart is highly active in energy production and consumption to support its contractile function, TRIM72 is an excellent model gene for studying the role of *cis*-elements in the evolution of tissue-specific gene expression and the functional consequences.

In this study, we investigate the evolution of the cardiac expression level of *TRIM72* in hominids and Old World monkeys. We use local-clock models (Yoder and Yang 2000) to estimate the evolutionary rates in major lineages of the primate phylogeny in which phenotypic changes are expected to have occurred, and conduct a test to examine possible rate acceleration in the promoter in the lineage ancestral to Old World monkeys. Using luciferase assay, transgenic reporter assay, and CRIPSR/Cas9-mediated genome editing, we identify two regulatory variants within the binding sites for NKX2-5 and TBX5 that affect *TRIM72* expression in the heart. We show that TRIM72 acts as a transcription factor regulating the expression of genes related to oxidative phosphorylation in mitochondria. Across the mammals, we show significant correlation between *TRIM72* expression and heart rate or body mass, suggesting that the heart-specific expression change of *TRIM72* may be a physiological adaptation to the changed heart rate or body mass.

## Results

### *TRIM72* Expression in the Heart Differs among Primate Species

First, we note that TRIM72 is a highly conserved protein. Across all primate species, the coding region (CDS) consists of six exons and 477 codons, with no need for indels in the alignment (supplementary fig. S1, Supplementary Material online). Fitting the M0 model of codon substitution (Goldman and Yang 1994; Yang 2007) to the alignment of the coding sequences from 13 primate species produced the maximum likelihood estimate (MLE) of the *d*_N_/*d*_S_ ratio to be 0.039 ± 0.009. Thus nonsynonymous mutations on average have a chance of fixation only 4% of that for synonymous mutations, indicating extreme evolutionary conservation of the protein.

**Fig. 1.**
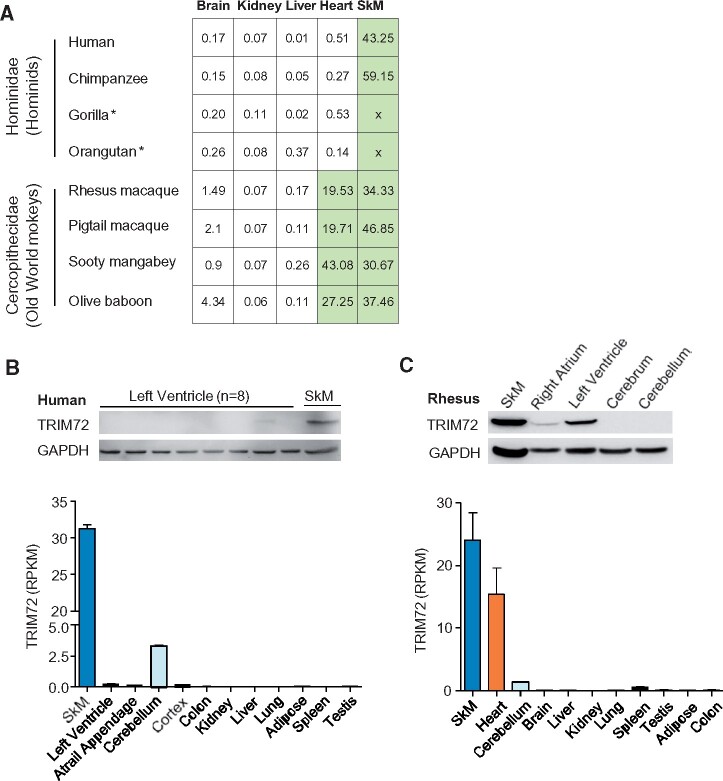
Different expression levels of *TRIM72* in the heart of hominids and Old World monkeys. (*A*) The expression patterns of *TRIM72* in hominids and Old World monkeys at the mRNA level (FPKM). Gorilla and orangutan data are from Brawand et al. (2011), and their skeletal muscle data are unavailable. Other data are from Pipes et al. (2013). (*B*) *TRIM72* is rarely expressed in human heart, whereas highly expressed in skeletal muscle. Upper panel: representative western blots of TRIM72. Lower panel: mRNA expression data from GTEx(V6p) (GTEx Consortium 2013) (see also supplementary fig. S2, Supplementary Material online). (*C*) *TRIM72* is highly expressed in the heart and skeletal muscle of rhesus macaque. Upper panel: representative western blots. Lower panel: expression levels of mRNA from RhesusBase (heart, *n* = 4, http://rhesusbase.cbi.pku.edu.cn/, last accessed March 24, 2021; Zhang et al. 2014). SkM, skeletal muscle.

*TRIM72* is highly expressed in striated muscle of mammals (Song et al. 2013; Liu et al. 2015; Lemckert et al. 2016). However, the expression of *TRIM72* in the heart is minimal in hominids, but abundant in Old World monkeys (fig. 1*A*). TRIM72 (both mRNA and protein) was almost undetectable in the human heart (fig. 1*B* and supplementary fig. S2, Supplementary Material online), but highly expressed in the heart of rhesus monkeys (fig. 1*C*). Notably, *TRIM72* is robustly expressed in the skeletal muscle of humans and chimpanzees (fig. 1*A* and *B* and supplementary fig. S2, Supplementary Material online). Thus the changes in the heart-specific *TRIM72* expression between primate species are not due to the loss or gain of the *TRIM72* gene, but are likely to be caused by *cis*-regulatory variants (such as mutations in the promoter).

### *TRIM72* Promoter Activity in the Heart Differs between the Human and Rhesus Monkey

Consistent with the differential expression of *TRIM72* in the human heart versus skeletal muscle, epigenetic modifications near the transcription start site (TSS) of the *TRIM72* locus displayed strong signals for enhancer/promoter activity (H3K27ac, H3K4me1, H3K4me3, and DNase I hypersensitive peaks) (Kim and Shiekhattar 2015) in skeletal muscle, which were markedly diminished in the heart (supplementary fig. S3, Supplementary Material online). Therefore, we hypothesized that the mutations in the promoter region might account for the heart-specific expression change of *TRIM72* in primates.

**Fig. 2.**
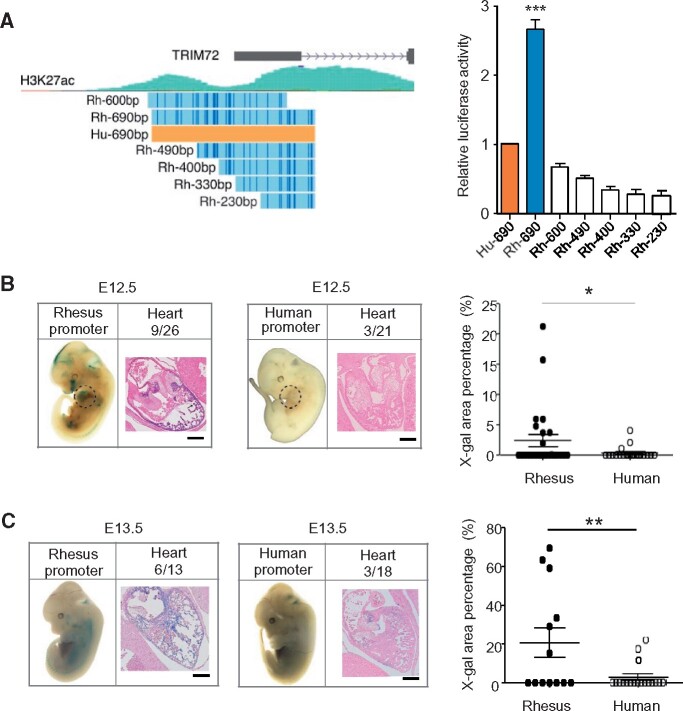
Different activity of *TRIM72* promoter between human and rhesus monkeys. (*A*) Comparison of *TRIM72* promoter activity of human versus rhesus using luciferase reporter assays in H9c2 cells (*n* = 8). Left panel, promoter fragments are aligned to hg19 genome by BLAT. Hu-690, human full-length promoter (chr16: 31224983–31225673, hg19); Rh-690, homologous region of Hu-690 in rhesus (chr20: 28866360–28867045, rheMac8). (*B*) In vivo transgenic analysis of human and rhesus *TRIM72* promoter activity in transgenic E12.5 mouse embryos (see also supplementary fig. S5, Supplementary Material online). (*C*) In vivo transgenic analysis of human and rhesus *TRIM72* promoter activity in transgenic E13.5 mouse embryos (see also supplementary fig. S6, Supplementary Material online). In *B* and *C*, left and middle panels: representative transgenic E12.5 and E13.5 mouse embryos and slices of the heart (Red, Eosin. Blue, X-gal. Scale bar, 200 μm). Numbers of embryos with LacZ positive hearts over the total number of transgenic embryos screened are indicated. Right panel: LacZ activity (mean ± SE) in the heart of transgenic embryos. *P < 0.05, **P < 0.01 (Student’s *t*-test).

To test this hypothesis, we performed luciferase reporter assays in H9c2 cardiomyoblasts to determine the promoter activity of rhesus and human *TRIM72*. As expected, human *TRIM72* promoter displayed relatively lower activity compared with the orthologous region of rhesus (fig. 2*A*). Furthermore, all truncations of the rhesus promoter showed diminished activity, indicating that the entire 690-bp promoter region (chr16: 31224983–31225673, hg19) is necessary for active transcription (fig. 2*A*). Therefore, we used this 690-bp promoter and its human orthologue as the *TRIM72* promoters in the subsequent experiments and analyses.

As *TRIM72* is highly expressed in the heart of E12.5 mouse embryos (supplementary fig. S4, Supplementary Material online), we also performed in vivo transgenic assays with a LacZ reporter driven by the 690-bp promoters. Indeed, the rhesus *TRIM72* promoter showed significantly higher activity than the human orthologue in mouse heart at E12.5 (fig. 2*B* and supplementary fig. S5, Supplementary Material online, LacZ positive hearts with the rhesus promoter was 34.6% vs. 14.3% with the human promoter, *P *=* *0.0383) and E13.5 (fig. 2*C* and supplementary fig. S6, Supplementary Material online, rhesus 46.1% vs. human 16.7%, *P *=* *0.006). The incomplete silencing of the 690-bp human *TRIM72* promoter in the murine heart may be attributable to other regulatory elements involved in the gene regulation as a result of enhancer redundancy (Osterwalder et al. 2018).

### Ancestral Reconstruction and Relative Rate Estimation Suggest Accelerated Evolution of the *TRIM72* Promoter in the Common Ancestor of Old World Monkeys

**Fig. 3.**
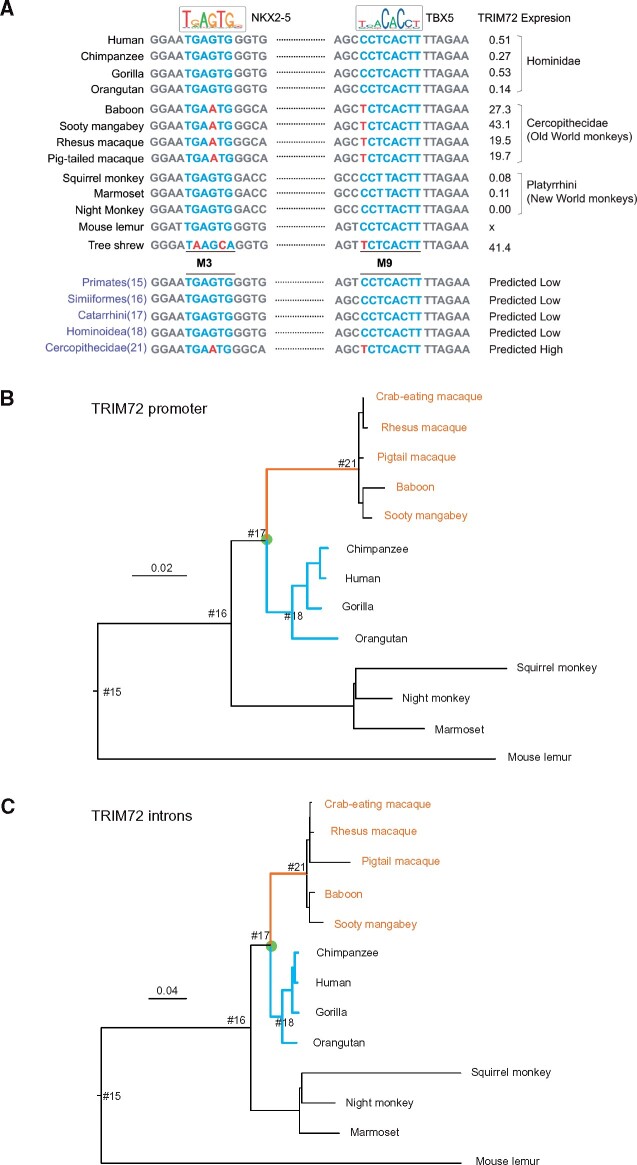
Ancestral reconstruction of the *TRIM72* promoter and estimation of relative evolutionary rates in primates. (*A*) Ancestral *TRIM72* promoter sequences at M3 and M9 reconstructed using maximum likelihood under the HKY+Γ_5_ model using the BASEML program in the PAML package. Blue indicates the transcription factor binding sites and red nucleotides indicate mutations that change the binding affinity of corresponding trans-factors. The cardiac expression level of *TRIM72* is given next to the sequence (data is missing for mouse lemur). (*B* and *C*) Species trees for *TRIM72* promoter (*B*) and introns (*C*) showing branch lengths estimated under the HKY+Γ_5_ model. The trees are unrooted but the root is placed on the mouse lemur branch for clarity. The branch ancestral to the Old World Monkeys (Cercopithecidae) is highlighted in blue, whereas the Hominid clade is in orange. The tree topology is used to fit the local-clock models which assign different rates for the colored branches. The node names of the trees are numbered for simplicity. #15: Primates; #16: Simiiformes; #17: Catarrhini; #18: Hominoide; #21: Cercopithecidae.

To estimate the ancestral state of *TRIM72* expression in the heart of primates, we reconstructed ancestral promoter sequences of hominids (Hominoidea) and Old World monkeys (Cercopithecidae) using the ML method implemented in the BASEML program in the PAML package (Yang 2007). The species phylogeny (supplementary fig. S7, Supplementary Material online) and the HKY+Γ_5_ model of nucleotide substitution were assumed. The ancestors should have high cardiac *TRIM72* expression if the reconstructed promoter sequences are similar to those in Old World monkeys, especially at the important sites identified below as M3 and M9 (see below). The reconstruction indicates that cardiac expression of *TRIM72* is high in the ancestors of Cercopithecidae, and is low in other primate ancestors, including Hominoidea, Catarrhini, and Simiiformes (fig. 3*A*). In other words, low cardiac *TRIM72* expression is the ancestral state and the common ancestor of Old World monkeys acquired the derived state of high expression, possibly through a burst of evolution in the promoter along the ancestral branch.

We tested for possible evolutionary rate acceleration in the promoter along the branch ancestral to the Old World monkeys. Branch lengths estimated by ML trees without assuming the molecular clock indicate clear violation of the molecular clock, with a characteristic hominoid slow-down. We fitted local-clock models (Yoder and Yang 2000) implemented in the BASEML program in the PAML package (Yang 2007) to sequence data for the promoter and the introns of 13 primate species (fig. 3*B* and *C*). We assigned three rates to the branches on the tree: *r*_1_ for the branch ancestral to the Old World monkeys (Cercopithecidae), *r*_2_ for the hominoids to account for hominoid slow-down, and a background rate *r*_0_ = 1 for all other branches. We tested the null hypothesis that the promoter rate and the intron rate on the focal branch are reduced by the same amount relative to the background rate; in other words, by using the intron rate as the reference, we tested the null hypothesis that the promoter rate mimics the intron rate without any acceleration (that is, $r_{1}^{\text{promoter}}/r_{1}^{\text{intron}}$ = 1). The test accounts for possible rate difference between the promoter and the introns and for violation of the strict molecular clock, such as the hominoid slow-down.

Relative to the background rate, there is comparable hominoid rate reduction in the promoter and in the introns, with *r*_2_ = 0.3525 ± 0.112 for the promoter and *r*_2_ = 0.216 ± 0.054 for the introns. Instead, the Cercopithecidae branch is much longer in the promoter tree than in the intron tree. The rate ratio is $r_{1}^{\text{promoter}}/r_{1}^{\text{intron}}$ = 0.527281/0.29572 = 1.78304, suggesting that relative to the background rate slowdown suggested by the introns, the Cercopithecidae branch had a rate that is 78% higher than expected if the promoter is following the rate trajectories of the introns. This is consistent with the hypothesis that the promoter experienced adaptive evolution along that branch, reflecting changes in cardiac expression of *TRIM72*. We conducted a further test of the null hypothesis that $r_{1}^{\text{promoter}}/r_{1}^{\text{intron}}$ = 1. We calculated the variances of the estimates using the delta approximation (Yang 2014), from which the approximate standard error of the rate ratio was 0.68907, giving the *P*-value as *Φ*(–0.78304/(1.96 × 0.68907)) = 0.28. Alternatively, similar calculation applying a normal approximation to the rate difference $r_{1}^{\text{promoter}}-r_{1}^{\text{intron}}$ gave the *P*-value 0.26. Thus, the evidence for promoter rate acceleration along the Cercopithecidae branch has not reached the level of statistical significance, when hominoid slowdown is accommodated in the model.

We note that such tests often lack power. Given the challenges of using statistical comparison of between-species variations to distinguish neutral and adaptive evolution (see Introduction), we consider the phylogenetic comparison as a hypothesis-generating exercise, and rely on experimentation to verify the functional significance and adaptive potential of mutations in the *TRIM72* promoter.

### Promoter Mutations Underlie Changes in Heart-Specific Expression of *TRIM72* in Primates

**Fig. 4.**
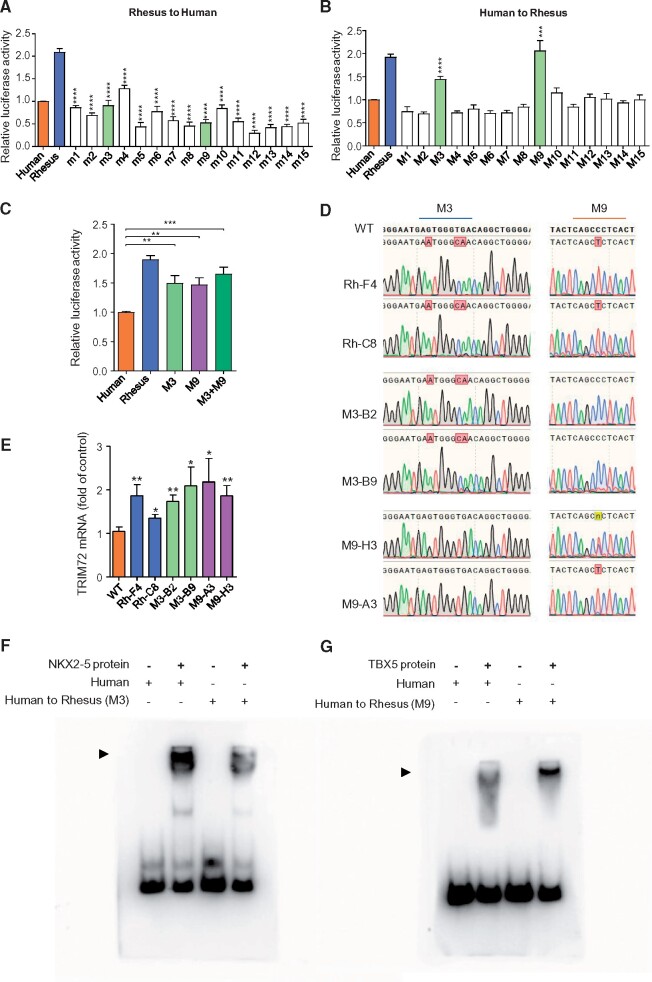
Mutations in the promoter contribute to the heart-specific expression change of *TRIM72* in primates. (*A*) All human-specific mutations reduced the promoter activity of rhesus *TRIM72* (*n* ≥ 6, mutation vs. rhesus as control). m1–m15, the Rhesus *TRIM72* promoters (Rh-690) with one of the 15 rhesus-to-human mutations. The reporter activities of m1–m15 were compared with that of Rhesus *TRIM72* promoter (Rh-690). Position of the mutations is shown in supplementary figure S8, Supplementary Material online. (*B*) Only two rhesus-specific mutations increased the promoter activity of human *TRIM72* (*n* ≥ 6, mutation vs. human as control). M1–M15 indicate the human *TRIM72* promoters (Hu-690) with one of the 15 human-to-rhesus mutations. The activities of M1–M15 were compared with human full-length promoter (Human Hu-690). Position of the mutations is shown in supplementary figure S8, Supplementary Material online. (*C*) Combination of M3 and M9 could restore the activity of human *TRIM72* promoter to the rhesus level. M3, M9, and M3+M9, human full-length promoter with human-to-rhesus mutation 3, 9, and both 3 and 9, respectively. (*D*) Sanger sequencing of *TRIM72* promoter region in edited hESC colonies. Sequence alignments of mutations 3 and 9 regions are shown. For each type of mutation, two clonal cell lines were expanded and tested. WT, promoter unmodified; Rh-F4 and Rh-C8, clones with *TRIM72* promoter replaced with rhesus ortholog; M3-B2 and M3-B9, clones of M3; M9-H3 and M9-A3, clones of M9 (see also supplementary fig. S9, Supplementary Material online, for genome editing strategy). (*E*) Replacement in human *TRIM72* promoter with rhesus-specific mutations upregulates *TRIM72* mRNA expression in hESC-derived cardiomyocytes (*n* ≥ 6, mutation vs. wild type control). (*F*) EMSA result showing human-to-rhesus M3 decreases the binding affinity of NKX2-5 to *TRIM72* promoter. The arrowhead indicates the protein-DNA complex of NKX2-5 and oligo probes. (*G*) EMSA result showing human-to-rhesus M9 increases the binding affinity of TBX5 to *TRIM72* promoter. The arrowhead indicates the protein-DNA complex of TBX5 and oligo probes. In panels *A*–*C* and *E*, *P < 0.05, **P < 0.01, ***P< 0.005, ****P< 0.001 (Student’s *t*-test).

To pinpoint specific mutations within the *TRIM72* promoter that may be responsible for the change of transcriptional activity in primates, we identified clade-specific mutations through multiple alignment of *TRIM72* promoters from eight primate species and examined their influence on promoter activity using luciferase assays (supplementary fig. S8, Supplementary Material online). Although all rhesus-to-human substitutions down-regulated the promoter activity, only mutations 3 and 9 (M3 and M9) in the reciprocal human-to-rhesus substitutions could raise the promoter activity of human *TRIM72* to the rhesus level (fig. 4*A*–*C*). To verify this result, we replaced the 690-bp human promoter sequence with its rhesus orthologue or just the M3 or M9 mutations in human embryonic stem cells (hESC) (fig. 4*D* and supplementary fig. S9, Supplementary Material online). After differentiating these genome-edited cells into cardiomyocytes (supplementary fig. S9*C*, Supplementary Material online), we determined the mRNA levels of *TRIM72* by RT-qPCR. Notably, the 690-bp rhesus promoter replacement and the two human-to-rhesus substitutions upregulated the expression of *TRIM72* compared with normal hESC-derived cardiomyocytes (fig. 4*E*). These results indicate that M3 and M9 are both necessary and sufficient for *TRIM72* promoter activity in the heart.

In silico scanning of potential transcription factors at these two sites identified an NKX2-5 binding site at M3 and a TBX5 binding site at M9 (supplementary table S1, Supplementary Material online). NKX2-5 and TBX5 are critical developmental transcription factors that regulate cardiac gene expression and vertebrate heart evolution (Olson 2006; Koshiba-Takeuchi et al. 2009; He et al. 2011; Dupays et al. 2015; Waldron et al. 2016; Steimle and Moskowitz 2017; Anderson et al. 2018). Using electrophoretic mobility shift assay (EMSA), we confirmed that the human M3 site is indeed the binding site of NKX2-5 and the mutation of M3 to rhesus greatly attenuated the recruitment of NKX2-5 (fig. 4*F*). In contrast, mutating human M9 to rhesus increased the binding affinity of TBX5 (fig. 4*G*). Therefore, the M3 and M9 mutations affect the recruitment of transcription factors to *TRIM72* promoter in primates. Importantly, NKX2-5 and TBX5 are predominantly expressed in the heart (supplementary fig. S10, Supplementary Material online), allowing for modulation of *TRIM72* expression specifically in myocardium without affecting its function in skeletal muscle.

### TRIM72 Influences the Cardiac Capacity by Controlling the Expression of Oxidative Phosphorylation-related Genes

**Fig. 5.**
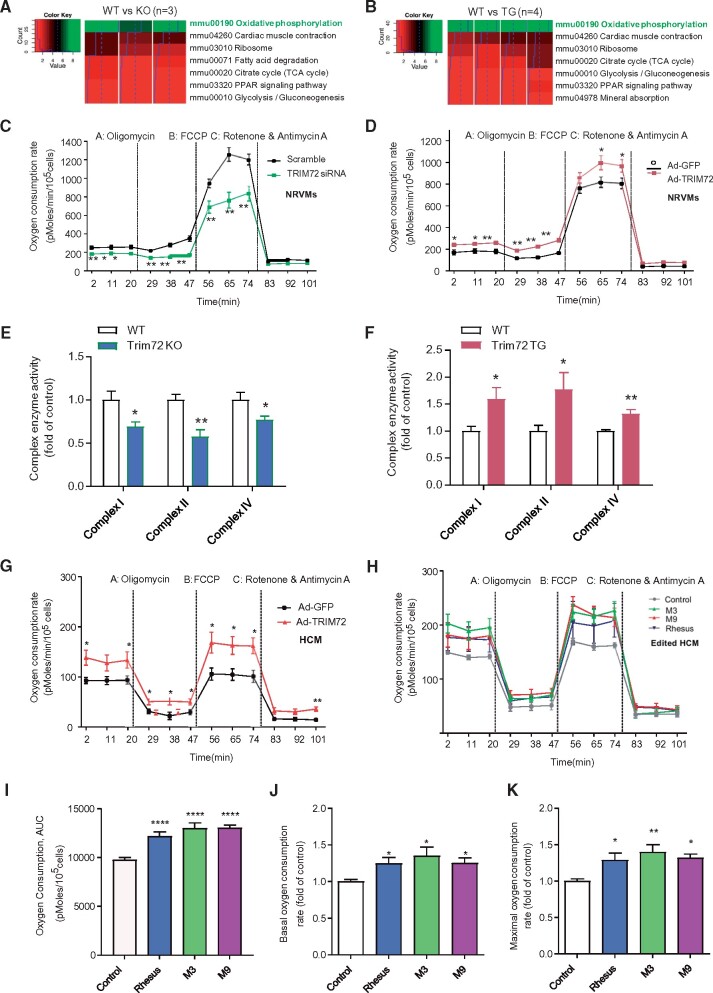
TRIM72 regulates mitochondrial respiratory capacity in cardiomyocytes. (*A* and *B*) KEGG pathway analysis showing that oxidative phosphorylation pathway is significantly altered in the *Trim72* knockout (*A*, *n* = 3 for each group) or transgenic hearts (*B*, *n* = 4 for each group). (*C* and *D*) Knockdown (*C*) or overexpression (*D*) of TRIM72 affects mitochondrial respiration in NRVMs (*n* ≥ 6). Oligomycin is an antibiotic that inhibits ATP synthase by blocking its proton channel (F0 subunit). FCCP (carbonyl cyanide-4-(trifluoromethoxy) phenylhydrazone) is a potent uncoupler of oxidative phosphorylation. Rotenone is an inhibitor of complex I and antimycin A is an inhibitor of complex III in electron transport chain. NRVMs: neonatal rat ventricular myocytes. OCR, oxygen consumption rate; Ad-GFP, adenovirus expressing GFP; Ad-TRIM72, adenovirus expressing TRIM72. (*E*) Reduced enzyme activity of mitochondrial respiratory complex in cardiomyocytes from *Trim72* knockout mice. (*F*) Increased enzyme activity of mitochondrial respiratory complex in cardiomyocytes from *Trim72* transgenic mice. (*G*) Overexpression of TRIM72 upregulates mitochondrial respiration in hESC-derived cardiomyocytes (HCM, *n* = 6). (*H*) *TRIM72* promoter mutations (Rhesus orthologue, M3 and M9) upregulate OCR in hESC-derived cardiomyocytes (*n* = 4–5). Edited HCM, *TRIM72* promoter modified hESC-derived cardiomyocytes. (*I*–*K*) Area Under Curve (AUC) of panel H, showing that *TRIM72* promoter mutations (Rhesus orthologue replacement, M3, and M9) upregulate total mitochondrial respiration (*I*), basal mitochondrial respiration (*J*), and maximal mitochondrial respiration (*K*) in hESC-derived cardiomyocytes (*n* = 4–5). In *C*–*G* and *I*–*K*, data are presented as mean ± SE. *P < 0.05, **P < 0.01, ***P < 0.001, ****P < 0.0001 (one-way ANOVA with post hoc *t*-test).

To investigate the functional consequences of *TRIM72* expression changes in the heart, we performed RNA-seq analysis of heart tissues from *TRIM72* transgenic or knockout mice and their corresponding wild type controls (Song et al. 2013; Liu et al. 2015). Pathway analysis revealed that one of the most significantly affected gene network was the oxidative phosphorylation pathway (fig. 5*A* and *B*). To explore the effects of TRIM72 on mitochondria, we measured mitochondrial oxygen consumption rate (OCR), which reflects mitochondrial function, in neonatal rat ventricular myocytes (NRVMs). Knockdown of TRIM72 markedly attenuated the respiratory capacity (fig. 5*C*), whereas overexpression of TRIM72 increased OCR (fig. 5*D*). Moreover, the enzyme activities of mitochondrial respiratory complexes were declined in the *TRIM72* deficient mouse heart (fig. 5*E*), whereas in the heart of *TRIM72* transgenic mice, the enzyme activities were significantly enhanced (fig. 5*F*). Consistently, overexpression of TRIM72 also upregulate the OCR in cardiomyocytes derived from hESC (fig. 5*G*). Thus, the gain or loss of *TRIM72* expression in the heart contributes to the respiratory capacity of mitochondria. To verify the effects of *TRIM72* promoter mutations on mitochondrial respiration, we measured the OCR of the cardiomyocytes differentiated from the genome-edited hESC (fig. 4*D*). Notably, the 690-bp rhesus promoter replacement and the two human-to-rhesus substitutions significantly increased the OCR as compared with that of the normal hESC-derived cardiomyocytes (fig. 5*H*–*K*), demonstrating that M3 and M9 can regulate the expression of *TRIM72* and influence the mitochondrial respiration of cardiomyocytes in primates.

**Fig. 6.**
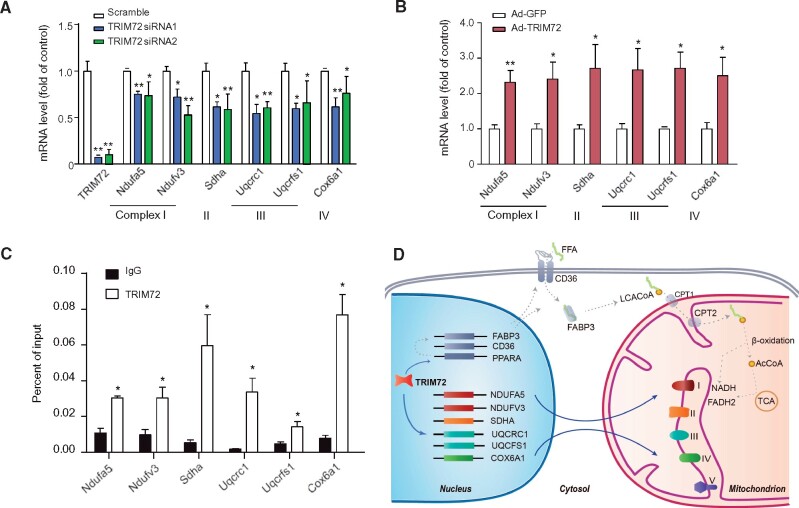
TRIM72 regulates the expression of oxidative phosphorylation-related genes at transcriptional level. (*A*) Knockdown of TRIM72 downregulated the expression of oxidative phosphorylation-related genes at the mRNA level in NRVMs (*n* ≥ 3). (*B*) Overexpression of TRIM72 upregulated the expression of oxidative phosphorylation-related genes at the mRNA level in NRVMs (*n* ≥ 3). (*C*) ChIP-qPCR shows that TRIM72 binds to the promoter regions of genes related to oxidative phosphorylation. (*D*) Schematic shows how TRIM72 regulates mitochondrial metabolism. TRIM72 influences the mitochondrial efficiency by regulating the expression of respiration complex-related genes, and by affecting lipid absorption via regulating the expression of PPARα (Liu et al. 2015). Ndufa5 and Ndufv3 are subunits of NADH dehydrogenase (Complex I). Sdha is a subunit of succinate dehydrogenase (Complex II), Uqcrfs1 and Uqcrc1 are subunits of cytochrome bc1 complex (Complex III), and Cox6a1 is a subunit of cytochrome c oxidase (Complex IV). Ad-GFP, adenovirus expressing GFP; Ad-TRIM72, adenovirus expressing TRIM72.

To investigate the mechanism underlying the TRIM72-induced metabolic changes in cardiomyocytes, we performed RT-qPCR and found that siRNA silencing of TRIM72 in NRVMs decreased the expression of the electron transport chain components Ndufa5, Ndufv3, Sdha, Uqcrc1, Uqcrfs1, and Cox6a1 (fig. 6*A*). In contrast, overexpression of TRIM72 augmented the expression of these genes (fig. 6*B*). Our previous studies have demonstrated that TRIM72 enhances cardiomyocyte lipid metabolism by upregulating PPARα at the transcriptional level (Liu et al. 2015). Using ChIP-qPCR, we found that TRIM72, in fact, was recruited to the promoter regions of these oxidative phosphorylation-related genes (fig. 6*C*). Taken together, these data demonstrate that TRIM72 regulates cardiac energy metabolism via modulating the transcription of the genes involved in not only substrate utilization but also mitochondrial oxidative phosphorylation, and thus contribute to the augmentation in cardiac metabolic capacity (fig. 6*D*).

### Differential Cardiac *TRIM72* Expression Is Correlated with Life-History Traits, Such as Heart Rate and Basal Metabolic Rate

An important question is what drives the changes in cardiac *TRIM72* expression during primate evolution. To address this question, we examined RNA-seq data of the heart from 29 mammalian species (supplementary fig. S11, Supplementary Material online). From the species phylogeny, the expression of *TRIM72* in the heart changed at least five times during mammalian evolution (supplementary fig. S11, Supplementary Material online). We confirmed the expression of *TRIM72* in the heart and skeletal muscle at both the protein and mRNA levels in six mammalian species (mouse, rat, rabbit, pig, sheep, and cattle) (supplementary fig. S12, Supplementary Material online). Simple correlation analysis using physiological measurements suggest that mammalian species with high cardiac *TRIM72* expression have high heart rate (*P *=* *0.0003), low body mass (*P *=* *0.0002), and high basal metabolic rate (BMR) per unit body weight (*P *=* *0.0052) (supplementary fig. S13*A*–*C* and table S2, Supplementary Material online). Moreover, among 7335 genes with well-annotated transcriptome in 20 mammalian species, *TRIM72* had the strongest correlation between the gene expression level and the heart rate (*R *=* *0.79, adjusted *P *=* *0.07) (supplementary fig. S13*D*, Supplementary Material online). Nevertheless, it is well-known that small mammals tend to have high heart rate and high BMR (supplementary fig. S14, Supplementary Material online), and such correlation may not suggest a cause–effect relationship. Furthermore, the correlation analysis ignores lack of independence due to phylogenetic relationships of the species.

To accommodate phylogenetic correlations, we used the phylogenetic logistic regression model of Ho and Ané (2014) to explore possible relationships between the cardiac *TRIM72* expression level and body mass or heart rate (supplementary fig. S15, Supplementary Material online). The tree for 29 mammalian species is used, with branch lengths estimated from VertLife (Upham et al. 2019) and approximate species divergence times retrieved from TimeTree (Kumar et al. 2017). The analysis suggested significant negative correlation between myocardial *TRIM72* expression and body mass, and positive correlation between *TRIM72* expression with heart rate.

## Discussion

Changes in gene regulatory networks (GRN) play important roles in animal phenotypic evolution (Wray 2007; Peter and Davidson 2011) and are often considered as the major genetic basis underlying primate evolution (King and Wilson 1975; Haygood et al. 2007; Blekhman et al. 2008; Prabhakar et al. 2008; Shibata et al. 2012; Boyd et al. 2015). Compared with protein-coding sequence changes which may have pleiotropic effects and are thus under strong purifying selection, *cis*-regulatory mutations can regulate gene expression in specific cell types at specific developmental stage and are thus under less selective constraint (Rodríguez-Trelles et al. 2003; Wray et al. 2003). Recent studies have identified a compendium of noncoding regions associated with cardiac functions and diseases (May et al. 2011; Dickel et al. 2016). However, the role of *cis*-regulatory variants in mammalian and primate heart evolution has not been well understood. In this paper we have used a combination of evolutionary analysis and experimental validation to investigate the role of mutations in the *TRIM72* promoter in the heart-specific expression of *TRIM72* among primate species and the potential contribution of *TRIM72* expression to the functional evolution of the heart in primates and mammals.

Our ancestral reconstruction analysis suggests that cardiac *TRIM72* expression was low in the ancestors, but became high along the branch ancestral to the Old World monkeys (fig. 3). Estimation of lineage-specific evolutionary rates using local-clock models suggests that the rate of promoter evolution along that branch was accelerated, indicating that the mutations in the promoter might be beneficial and were fixed by positive selection. Indeed, our functional assays showed that mutations in the promoter region (especially at the M3 and M9 sites) can account for the differences in the cardiac expression of *TRIM72* between the Old World monkeys (rhesus macaque) and the hominids (human). Furthermore, the presence of TRIM72 increases myocardial energy metabolism by regulating the expression of genes associated with energy substrate utilization (Liu et al. 2015) and mitochondrial oxidative phosphorylation (figs. 5 and 6). Importantly, perturbation of *TRIM72* expression changes oxygen consumption in rodents as well as humans, indicating highly conserved function of TRIM72 in regulating mitochondria metabolism (fig. 5). Mitochondria are the power plants supporting cardiomyocyte contraction, and is of vital importance in mammalian heart function and development (Zhou and Tian 2018; Zhao et al. 2019). The results provide strong evidence that *TRIM72* expression affects the metabolic capacity of the heart in mammals. The hypothesis is supported by our phylogenetic regression analysis on a broader timescale including different mammalian species, which suggests that high cardiac *TRIM72* expression is significantly correlated with high heart rate and small body size (supplementary fig. S15, Supplementary Material online).

The mammalian species show a wide range of heart rate, and heart rate is strongly correlated with body mass and BMR, with smaller mammals having faster heartbeat and higher BMR (supplementary fig. S14, Supplementary Material online). When the body size of a species changes to adapt to new environments, the cardiovascular system needs to evolve accordingly to ensure that the nutrient and oxygen supply can meet the metabolic demands of the species (Speakman 2005; Capellini et al. 2010; DeLong et al. 2010; Hillman and Hedrick 2015b). The major cardiovascular steps in the support of endothermy are the increased rates of blood flow and cardiac power output, achieved by elevated heart rates (Hedrick and Hillman 2016). Thus, to maintain a stable body temperature, small mammals need more cardiac energy production in cardiomyocytes to support their fast heart rate (Dobson 2003). It is a plausible hypothesis that changes in body size are a major driving factor for changes in cardiac *TRIM72* expression. Nevertheless, heart rate appears to be a polygenic trait (Nolte et al. 2017) and TRIM72 is likely one of the factors that contribute to cardiac capacity. Besides *TRIM72*, other genes whose expression levels show high correlation with heart rate, such as *BCL3, ABTB1, ZFP36, EFNB3*, *DPEP1*, *OGDHL* (supplementary fig. S13*D*, Supplementary Material online), are potentially involved in the change of cardiac capacity. The relative importance of those genes in the functional evolution of the heart across mammalian species merits further investigation.

## Materials and Methods

### Vector Construction

Human *TRIM72* promoter (chr16: 31224983–31225673, hg19) and rhesus *TRIM72* promoter (chr20: 28866360–28867045, rheMac8) were cloned using genomic DNA extracted from human VSMC cells and rhesus heart tissues.

For the in vitro luciferase reporter assay, original *TRIM72* promoters, promoters with truncations (supplementary table S3, Supplementary Material online) or point mutations (supplementary tables S4 and S5, Supplementary Material online) were cloned into pGL4.21 vector (E676A, Promega), and the SV40 enhancer was replaced with a *TRIM72* downstream element (chr16: 31226063–31226476, hg19). Mutation vectors were constructed with mutated primers using Gibson assembly (C215, Vazyme).

For the in vivo LacZ reporter assay, human and rhesus *TRIM72* promoters were cloned into a modified pWHERE vector (He et al. 2011) (H19-Hsp68 miniP-LacZ-ΔCpG NLS-H19, InvivoGen) using *Hin*dIII and *Xho*I. All constructs were Sanger sequenced using plasmid DNA isolated from transformed bacterial cells.

### Luciferase Assay

The rat cardiac myoblast cell line H9c2 (CRL-1446, ATCC) was used for luciferase reporter assays. The cells were plated in 24-well plates, and 200 ng firefly luciferase plasmid, 10 ng renilla luciferase plasmid and 0.6 µl ScreenFectA (S-3001, ScreenFect GmbH) were added to each well. 48 h post transfection, luciferase activity was determined using Dual-Luciferase Assay kit (E1910, Promega) according to manufacture instructions. The luminescence signal was detected in a white 96-well plate using Biotek Synergy HTX. The reporter gene activity of firefly luciferase was normalized to that of renilla luciferase to determine the activity of functional elements.

### Animals and Transgenic LacZ Reporter Assay

All animal procedures and euthanasia were performed in accordance with protocols approved by the Institutional Animal Care and Use Committee of Peking University, and conformed to the Guide for the Care and Use of Laboratory Animals (NIH publication No. 86-23, revised 1985). All mice were maintained in a temperature-controlled barrier facility with a 12-h light/dark cycle and were given free access to food and water in the Laboratory Animal Center at Peking University, Beijing, China (an AAALAC-accredited experimental animal facility). Only male animals were used in this study. The generation of MG53TG and MG53^–/–^ mice was described previously (Song et al. 2013).

Transgenic mice for in vivo reporter assay were generated by pronuclear injection. LacZ reporter plasmids with human or rhesus *TRIM72* promoter were linearized with *Pac*I and then injected into fertilized oocytes and transplanted into pseudopregnant ICR mice. Embryos were collected at indicated stages (E12.5 and E13.5) and stained for β-galactosidase activity. Mice carrying LacZ transgenes were confirmed by PCR using DNA extracted from yolk sacs. The genotyping primers were: LacZ-u, ATGAGAATGGCAACCCCTGG and LacZ-d, GAACTGTTGCTGGTGCTTGG.

For β-galactosidase staining, embryos were washed 2 times with ice cold PBS, fixed on ice for 60 min in fixing buffer (2% paraformaldehyde, 0.2% glutaraldehyde in PBS, pH 7.4), washed 2 times for 15 min with ice-cold PBS at RT, and stained overnight at RT in staining buffer (5 mM K_4_Fe(CN)_6_, 5 mM K_3_Fe(CN)_6_, 2 mM MgCl_2_, 0.02% NP-40, and 1 mg/ml X-gal). The next day, embryos were washed 3 times for 10 min with PBS at RT. Then, the embryos were fixed again using fixing buffer for 4 h at RT and stored at 4 °C. These LacZ positive embryos were imaged using a Leica M205 C microscope with LED3000 RL.

For detailed section analyses, X-Gal-positive embryos were embedded in paraffin using a standard protocol (He et al. 2011), and sagittal sections with thickness of 5 μm were counterstained with eosin for visualization of embryonic structures. The heart sections were photographed using an Axio Scope.A1 (Zeiss) microscope.

For X-gal staining analyses, the X-gal positive area and whole heart area were quantified using ImageJ software (Rueden et al. 2017); X-gal area percentage was defined as the ratio of these two values.

### DAB Staining

Wild type E12.5 and E13.5 mouse embryos were collected and fixed using fixing buffer (2% paraformaldehyde, 0.2% glutaraldehyde in PBS, pH 7.4) at RT for 4 h and then embedded in paraffin wax. Sagittal sections were cut at a thickness of 5 μm followed by deparaffinization and antigen retrieval. The sections were blocked with 5% goat serum and incubated with normal mouse IgG (sc-2025, Santa Cruz) or custom made TRIM72 antibody overnight at 4 °C. Next, the embryo sections were stained with Pierce DAB Substrate Kit (Thermo) following manufacturer’s instructions. These slices are stained with hematoxylin after the DAB reaction. The sections were photographed using an Axio Scope.A1 (Zeiss) microscope.

### H1 ES Cells Culture and Cardiomyocyte Differentiation

The human H1 embryonic stem cells were maintained and differentiated in Matrigel (354277, Corning)-coated plates following a standard protocol (Lian et al. 2013).

### CRISPR/Cas9 Editing of *TRIM72* Promoter in H1 ES Cells

gRNAs were designed using online software from Feng Zhang’s lab (http://crispr.mit.edu/). gRNA1: CTGATGCC GAGTGATCAATG; gRNA2: ATGGGACTCTGACGGCCAAG.

The human *TRIM72* promoter region was amplified by PCR using primer pairs (5K-u2: GAGCACCAGCTTCCTGA GACTTT and 5k-d3: ATACCAACGAAAGGACGGTGGTC). PCR products were subcloned into T-vectors and served as donor templates for CRISPR/Cas9 mediated homologous recombination, containing the 5′arm (chr16: 31222399–31223506, hg19) and 3′arm (chr16: 31225890–31227513, hg19) of the human genomic sequence.

Human H1 embryonic stem cells were cultured to 80–90% confluence in mTeSR1 medium (85850, STEMCELL Technologies). Cells were then digested into single cells using Accutase (07920, STEMCELL Technologies) and resuspended in mTeSR1 + 5 µM Y27632. Cells (1 × 10^7^) were electroporated with a combination of Cas9 plasmids (8 μg) (#44719, Addgene), two gRNAs (5 μg each), and recombinant donor plasmid (8 μg) in 800 μl ice cold DPBS (Invitrogen) using the Gene Pulser Xcell System (Bio-Rad) at 250 V and 500 μF in 4-mm cuvettes (Bio-Rad). H1 clones were then picked and screened by PCR using the following primers: Primer4: CAGTTTCATTCTCTTGCATAAGG and Primer6: CTTAAGCTCCTCAGACCCTCTTTC. PCR products were Sanger sequenced to confirm the genomic sequence of the edited clones.

### Immunofluorescence Staining

Differentiated ES cells were plated on glass slides at an appropriate density. Then they were fixed with 4% paraformaldehyde (pH 7.4) for 10 min, permeabilized with 0.1% Triton dissolved in PBS for 10 min at RT, and incubated overnight in blocking solution containing antibody against NKX2-5 (sc-376565, Santa Cruz) or TNNT2 (MA5-12960, Invitrogen). A second antibody conjugated with TRITC or FITC were used for immunostaining. All immunofluorescence images were captured by a laser scanning confocal microscope system (LSM710, Zeiss).

### Reverse Transcript PCR and Real-Time Quantitative PCR

Total RNA was extracted from heart or skeletal muscle tissue with TRIzol reagent (Invitrogen), and 2 μg total RNA was reverse transcribed using MLV reverse transcriptase and oligo-dT (C1101, Promega). 2 μl cDNA and PrimeSTAR HS premix was used for reverse transcript PCR, and 10 μl of PCR products were analyzed using 1% agarose gels and imaged using a Tanon 1600 image system (Tanon). For real-time quantitative PCR, 2 μl diluted cDNA and SYBR Green mix (A301, GenStar) were used with an ABI Real-Time PCR System (Step One Plus, ABI). Primers used are listed in supplementary table S6 (for fig. 6*A* and *B*) and table S9 (for fig. S12), Supplementary Material online.

### Western Blotting

Fresh or frozen tissues were ground into powder in liquid nitrogen, and total protein was extracted using RIPA buffer (25 mM Tris–HCl pH 7.6, 150 mM NaCl, 1% NP-40, 1% sodium deoxycholate, 0.1% SDS) supplemented with Protease Inhibitor Cocktail (11697498001, Roche), and 1 mM PMSF (P7626, Sigma). The lysate was incubated at 4 °C for 1 h with rotation, then centrifuged at 13,000 × g for 15 min at 4 °C and the supernatant was recovered. 50 μg total protein was used for SDS-PAGE separation and analyzed by western blot using a custom made antibody against TRIM72, NKX2-5 (AF7575, Beyotime), TBX5 (13178-1-AP, Proteintech), GAPDH (AM4300, Invitrogen), or β-Tubulin (ab6046, Abcam). All secondary antibodies were horseradish peroxidase-conjugated. The blots were imaged using ChemiDoc MP (Bio-Rad).

### Electrophoretic Mobility Shift Assay

EMSAs were carried out using the lightshift chemiluminescent EMSA kit (Thermo Fisher) according to the manufacturer’s instruction. Briefly, 100 fmol of oligonucleotides containing M3 or M9 labeled with biotin at the 3′ end were incubated with NKX2-5 or TBX5 protein synthesized by TNT quick coupled transcription/translation system (Promega) in binding buffer (10 mM Hepes, PH 7.9, 50 mM KCl, 1 mM DTT, 2.5% glycerol, 5 mM MgCl_2_, 0.05% NP-40, 1 µg poly(dI-dC)) at room temperature for 20 min. Binding mixture was resolved on 5% polyacrylamide gel, then transferred electrophoretically to nylone membrane (Merck Millipore) and detected by horseradish peroxidase-conjugated streptavidin.

### RNA-Seq and Data Analysis

Total RNA was extracted from heart tissues of TRIM72 transgenic, knock-out, or wild type mice using RNeasy Fibrous Tissue Mini Kit (QIAGEN). RNA samples were collected from 3-4 hearts for each group. RNA-Seq libraries were prepared in BIOPIC at Peking University and sequenced on an Illumina HiSeq2000 system.

RNA-Seq reads were mapped to the mouse genome (mm9) by TopHat (v2.0.8) and Cufflinks (v2.1.1), according to computational pipelines as reported previously (Zhang et al. 2014; Liu et al. 2015). R package GAGE (v2.30.0) (Luo et al. 2009) was used for functional enrichment and KEGG pathway analyses.

### ChIP-qPCR and Data Analysis

NRVMs were cultured in DMEM supplemented with 10% FBS at 37 °C under 5% CO_2_. About 1 × 10^7^ cells were cross-linked at room temperature for 10 min in 1% formaldehyde followed by quenching in 125 mM glycine. The cells were collected and washed twice with PBS, then incubated in 1 ml nuclei isolation buffer (10 mM HEPES, pH 7.9, 85 mM KCl, 1 mM EDTA, 0.5% NP-40) on ice for 10 min. Pellets were collected by centrifugation and resuspended in 200 μl lysis buffer (50 mM Tris-Cl, pH 8.0, 0.5% SDS, 10 mM EDTA, 0.5 mM EGTA). Sonication was performed with a BioRuptor (Diagenode; 24 cycles, 30 s on, 90 s off, high-power). Undissolved chromatin was discarded by centrifugation at 15,000 × g at 4 °C for 10 min. 10 μl supernatant was saved as input. The other supernatant was diluted with 800 μl dilution buffer (20 mM Tris-Cl, pH 8.0, 150 mM NaCl, 2 mM EDTA, 1% Triton) and incubated with 5 μg antibody and 20 μl protein A Dynabeads (10001D, Invitrogen) overnight at 4 °C with rotation. Beads was sequentially washed with 1 ml of washing buffer I (20 mM Tris-Cl, pH 7.4, 150 mM NaCl, 0.1% SDS, 1% Triton, 2 mM EDTA), washing buffer II (20 mM Tris-Cl, pH 7.4, 250 mM NaCl, 0.1% SDS, 1% Triton, 2 mM EDTA), washing buffer III (10 mM Tris-Cl, pH 7.4, 250 mM LiCl, 1% NP-40, 0.7% Deoxycholate, 1 mM EDTA), and washing buffer IV (10 mM Tris-Cl, pH 8.0, 1 mM EDTA). All buffers contained Protease Inhibitor Cocktail (11697498001, Roche) and 1 mM PMSF (P7626, Sigma). ChIP complexes were eluted with 150 μl elution buffer (50 mM Tris-Cl, pH 8.0, 10 mM EDTA, 1% SDS, 0.5 mg/ml proteinase K) and cross-links were reversed by incubation at 65 °C overnight. DNA was purified with phenol chloroform extraction and ethanol precipitation. RNA was degraded by RNase A (R4642, Sigma) at 37 °C for 1 h. Then, DNA was repurified by phenol: chloroform: isoamyl alcohol extraction and ethanol precipitation. The immunoprecipitated DNA and input DNA were quantified by qPCR analysis using primers listed in supplementary table S7, Supplementary Material online. ChIP-enrichment was calculated by normalizing the specific antibody-enriched or the nonspecific IgG-enriched chromatins to input DNA.

### Oxygen Consumption Assay

OCR was measured in NRVMs using Seahorse XF24 Flux analyzer (Agilent Technologies) following the instructions of Seahorse XF Cell Mito Stress Test Kit with slight modifications as previously described (Wang et al. 2017).

Briefly, NRVMs or hESC derived cardiac myocytes were seeded into a XF24 cell culture microplate at 1 × 10^5^ cells per well. For TRIM72 overexpression or knockdown, cells were infected with TRIM72 adenovirus or transfected with TRIM72 siRNA for 48 h. Before the assay, cells were incubated in unbuffered medium (DMEM) (D5030, Sigma) supplemented with 32 mM NaCl, 25 mM glucose and 2.5 mM sodium pyruvate, pH 7.4) for 1 h at 37 °C without CO_2_. Then basal respiration was assessed in untreated cells. ATP turnover was assessed after adding 1 µM Oligomycin (ab141829, Abcam). Maximum respiratory capacity was determined by adding 1 µM arbonyl cyanide 4-(trifluoromethoxy) phenylhydrazone (FCCP). Finally, nonmitochondrial respiration was measured after adding 1 µM Rotenone (R8875, Sigma) and 1 µM Antimycin A (ab141904, Abcam). Upon completion of the measurement, the OCR value (pMoles/min) was normalized with the total protein amount of each well and used for further analysis.

### Custom-Made TRIM72 Antibody Validation

To validate that the custom-made antibody against TRIM72 could be used in different species, TRIM72 coding sequences of mouse, rat, rabbit, sheep, pig, cow, rhesus, and human were cloned using cDNAs from respective skeletal muscle tissues. These coding sequences were cloned into pEASY-Blunt M2 vector (Transgen), which has a CMV promoter and a Myc tag at the C terminus. The plasmids were Sanger sequenced and expressed in HEK293T cells. The overexpressed TRIM72 proteins were detected using the custom TRIM72 antibody and confirmed by anti-Myc antibody.

### Alignment and Phylogenetic Analysis of the CDS, Promoter, and Intron Data

There are seven exons and six introns in the *TRIM72* gene (supplementary fig. S1, Supplementary Material online). We retrieved the CDS and promoter region of *TRIM72* for 13 primate or 29 mammal species from GenBank or Ensembl. We retrieved the intron sequences from 13 primate species only, as the sequences from different mammals are too divergent for the alignment to be reliable. Many insertions/deletions were found in the CDS of squirrel monkey and pigtail macaque, apparently because the CDS was based on predictions with inaccurate splicing sites and annotation errors. Thus, we downloaded the heart or skeletal muscle RNA-seq data for those species (supplementary table S2, Supplementary Material online), and aligned the raw reads to the *TRIM72* gene of each species and then assembled the CDS based on the aligned RNA-seq reads. All 13 primate species had 1434 nucleotides (477 codons), with no indels in the CDS alignment.

Maximum likelihood trees inferred using the exon, intron, and the promoter sequence alignments were largely consistent with well-accepted mammalian phylogenies. We focus on evolutionary rates rather than the species tree topology, and thus used the accepted species tree as given in our analysis. We reconstructed ancestral sequences for the promoter under the HKY+Γ_5_ model (Yang et al. 1995), using the BASEML program in the PAML package (Yang 2007). We applied local-clock models (Yoder and Yang 2000) implemented in BASEML to the promoter and intron alignments for the 13 primate species (fig. 3). The models allow us to estimate relative rates for branches on the tree while accommodating well-known violation of the molecular clock, such as the Hominoid slowdown. We tested the null hypothesis that the promoter and the intron rates for the branch ancestral to Old World Monkeys are reduced by the same amount, in effect using the introns as reference to test for possible rate acceleration in the promoter.

### Public RNA-Seq Data Collection and Analysis

We collected publicly available cardiac transcriptome data from 29 mammalian species from public databases (supplementary table S2, Supplementary Material online). The reference genome and GTF files for each species were downloaded from NCBI or other public database (Hu et al. 2012; Keane et al. 2015). The genome FASTA files were indexed using Bowtie2 (v 2.3.1) (Langmead and Salzberg 2012). The data were analyzed using Tophat (v2.1.1) (Kim et al. 2013) and Cufflinks (v2.2.1) (Trapnell et al. 2012).

### Tissue Samples

We obtained human heart ventricle samples from Dr Xiao Chen from Fu Wai Hospital; skeletal muscle samples from the Third Hospital of Peking University. All donors provided informed written consent. The study protocol conformed to the ethical guidelines of the 1975 Declaration of Helsinki and was approved by the Human Ethics Committee of Fu Wai Hospital.

Heart and skeletal muscle samples of mouse (*Mus musculus*), rat (*Rattus norvegicus*), and rabbit (*Oryctolagus cuniculus*) were obtained from the Laboratory Animal Center of Peking University. Heart and skeletal muscle samples of sheep (*Ovis aries*), pig (*Sus scrofa*), and cattle (*Bos taurus*) were obtained from local market, and the *TRIM72* open reading frames were cloned, sequenced and aligned with NCBI annotated sequences to validate the species.

*TRIM72* knock-out mice and heart-specific transgenic mice were generated and maintained as described (Song et al. 2013; Liu et al. 2015). All animal procedures were carried out in compliance with the protocols approved by the Institute of Animal Care and Use Committee of Peking University and in accordance with the Guide for the Care and Use of Laboratory Animals (National Institutes of Health publication No. 86-23, revised 1985).

## Supplementary Material

Supplementary data are available at *Molecular Biology and Evolution* online.

## Supplementary Material

msab083_Supplementary_Data

## Data Availability

The data underlying this article are available in its online supplementary material. The data for phylogenetic analysis of TRIM72 in primates are available in https://github.com/fengpku/MBE_data_2021; last accessed March 24, 2021.
