# Supplementary material for: Functional and Adaptive Significance of Promoter Mutations That Affect Divergent Myocardial Expressions of TRIM72 in Primates
Source: Mol Biol Evol. 2021 Mar 21;38(7):2930–45. doi: 10.1093/molbev/msab083 (PMC8233513; doi:10.1093/molbev/msab083)
Supplement: msab083_Supplementary_Data [file msab083_supplementary_data.pdf]

Figure S1

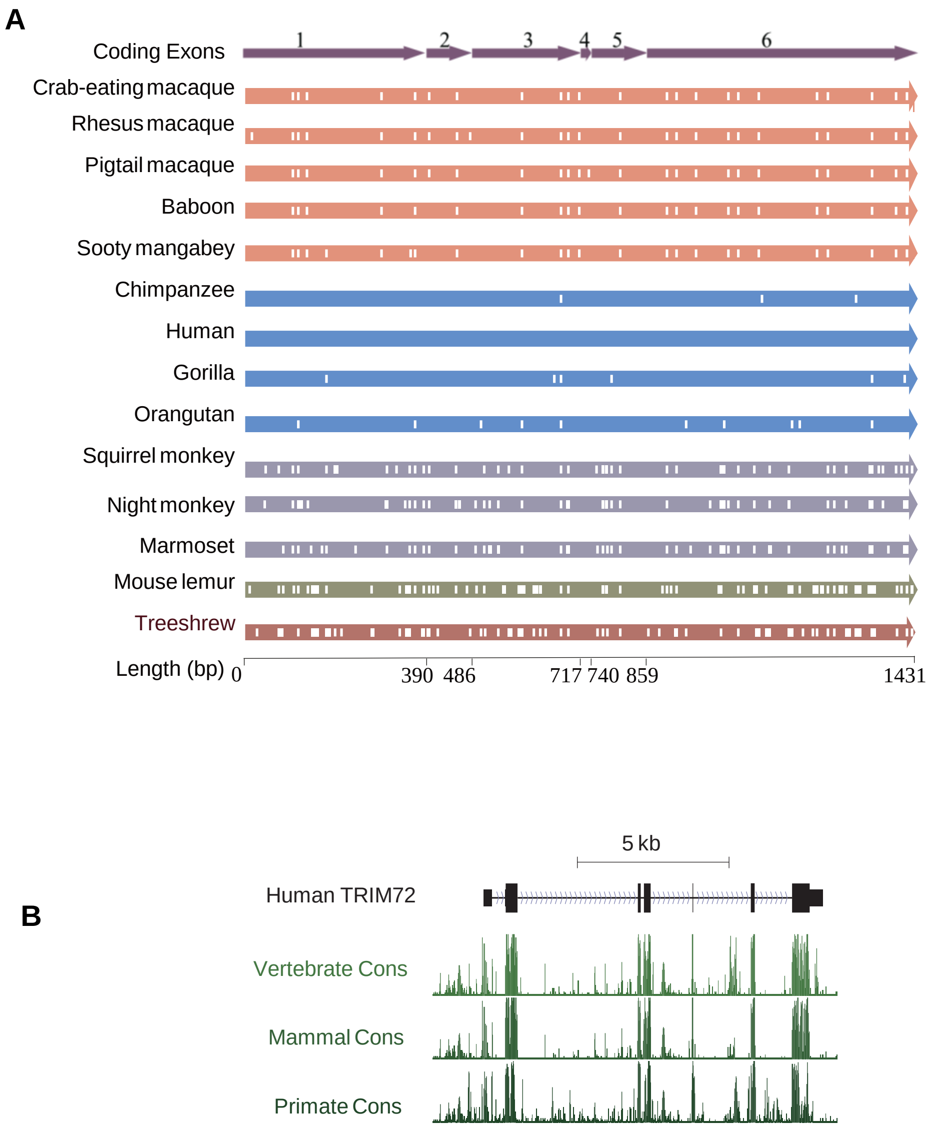

Figure S1. *TRIM72* CDS are conserved in mammalian species. (A) Multiple alignments of *TRIM72* CDS in 13 primate species. For each specie, *TRIM72* CDS was manually refined based on RNA sequencing data and its length is 1431bp in all 14 species shown. White dashes indicate sequence differences when using human *TRIM72* as reference. The exons are shown above the alignment. (B) *TRIM72* CDS is highly conserved across primates and mammals. Measurements of evolutionary conservation using phastCons from the PHAST package (green tracks). The figure was generated in UCSC genome browser.

Figure S2

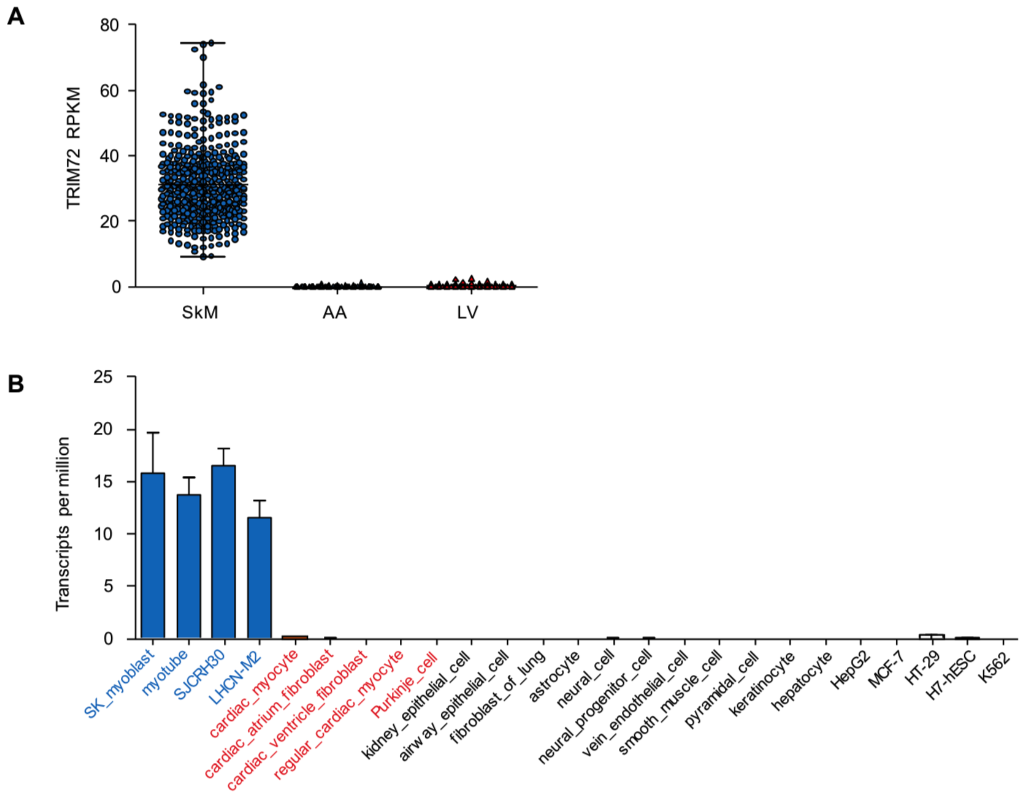

Figure S2. *TRIM72* is barely expressed in human hearts. (A) Scatter plot of *TRIM72* mRNA expression levels in the skeletal muscle and heart of human. SkM, skeletal muscle, n=430; AA, atrial appendage, n=194; LV, left ventricles, n=218. Data are from the GTEx database (V6p). (B) The expression levels of *TRIM72* mRNA in different human cell lines from ENCODE. Human skeletal muscle cell lines are marked in blue; human heart cell lines are highlighted in red. Related to Figure 1.

Figure S3

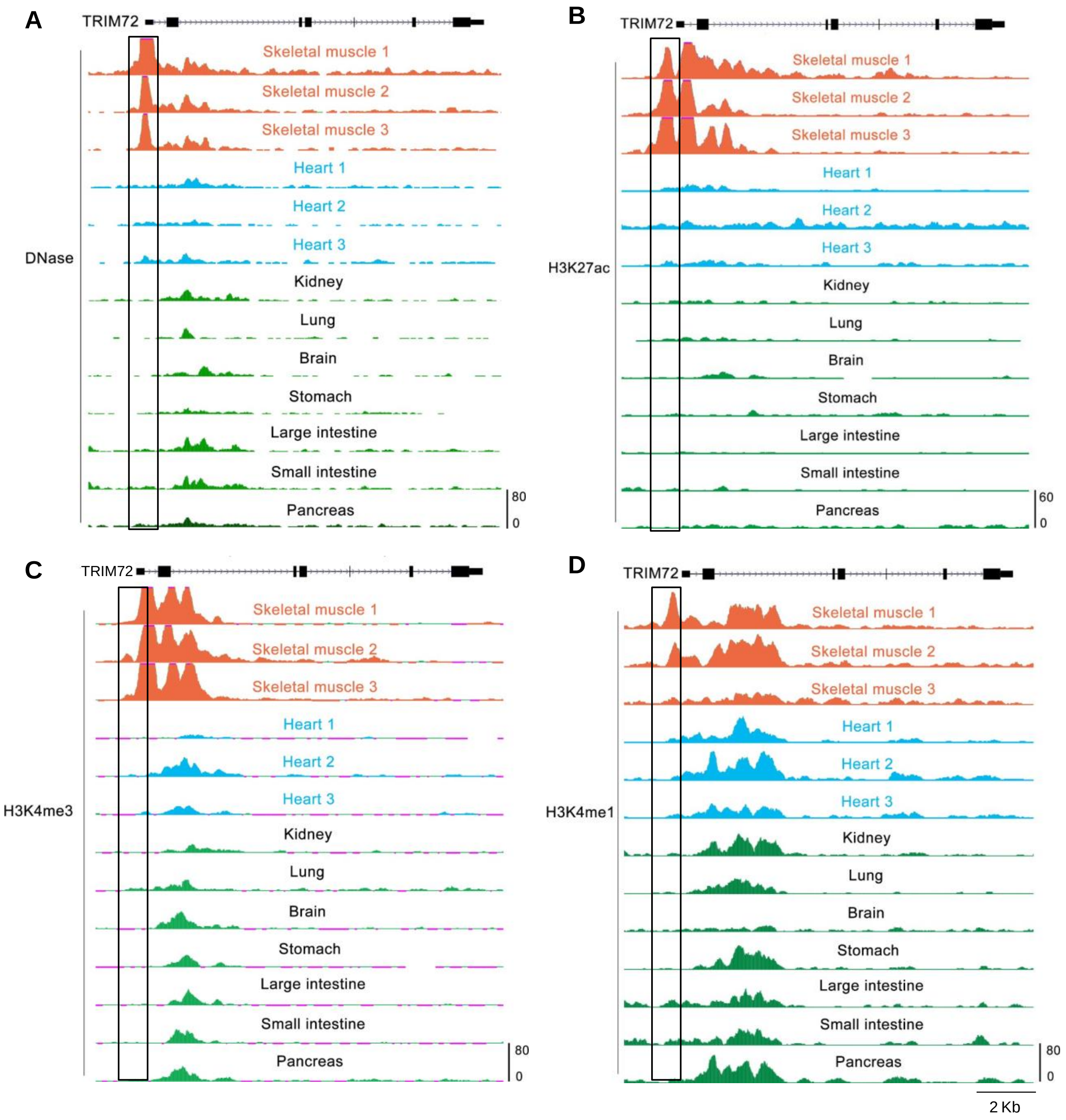

Figure S3. Epigenetic modifications indicate that *TRIM72* promoter activity is diminished in human heart. (A-D) DNase I hypersensitivity signals (chromatin accessibility), H3K27ac signals (transcription activity), H3K4me3 signals (promoter marker), H3K4me1 signals (enhancer marker) at *TRIM72* loci in various human tissues. Data are from Roadmap database. Epigenetic profiles of the skeletal muscle and heart tissues are shown in orange and blue, and others are shown in green. Dotted boxes indicate the promoter region of *TRIM72*. All signals were scaled to 80. Related to Figure 2A.

Figure S4

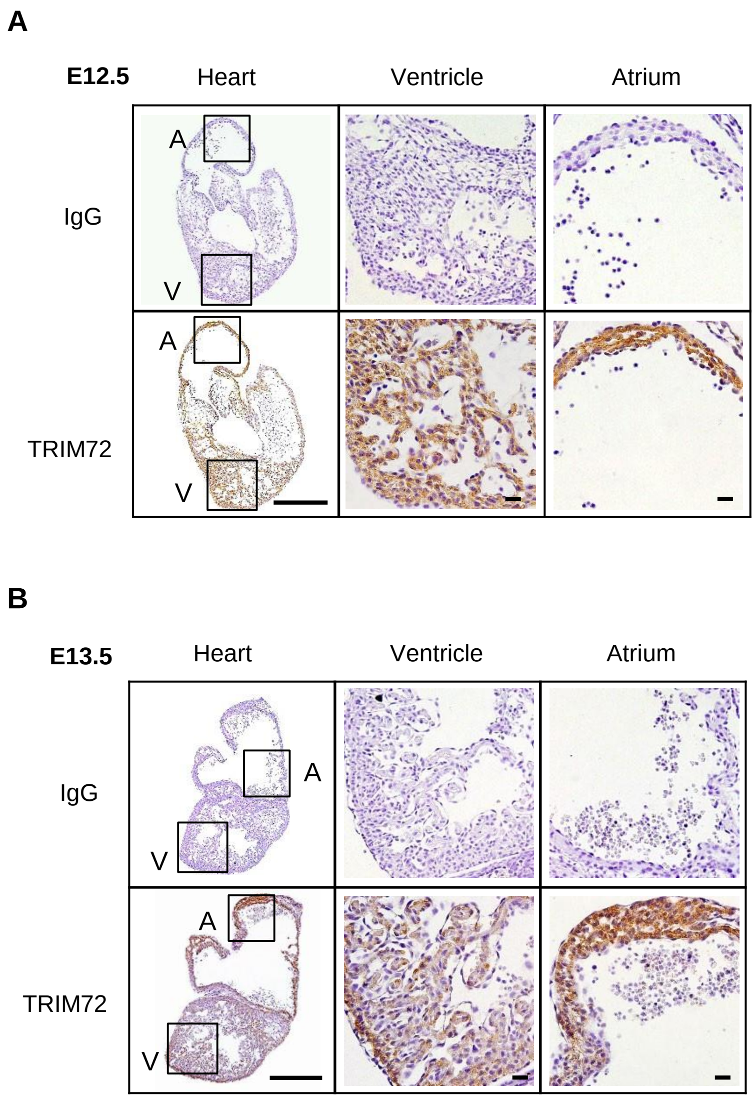

Figure S4. *TRIM72* is highly expressed in E12.5 and E13.5 mouse hearts. (A, B) DAB staining of TRIM72 in the hearts of wild type E12.5 (A) and E13.5 (B) embryos. Left panel: whole heart staining; Middle panel: enlarged view of boxed ventricle region; Right panel: enlarged view of boxed atrium region. A: atrium; V: Ventricle. Scale bars: left, 1 mm; middle and right, 25 μm. These slices are stained with hematoxylin after DAB reaction.

Figure S5

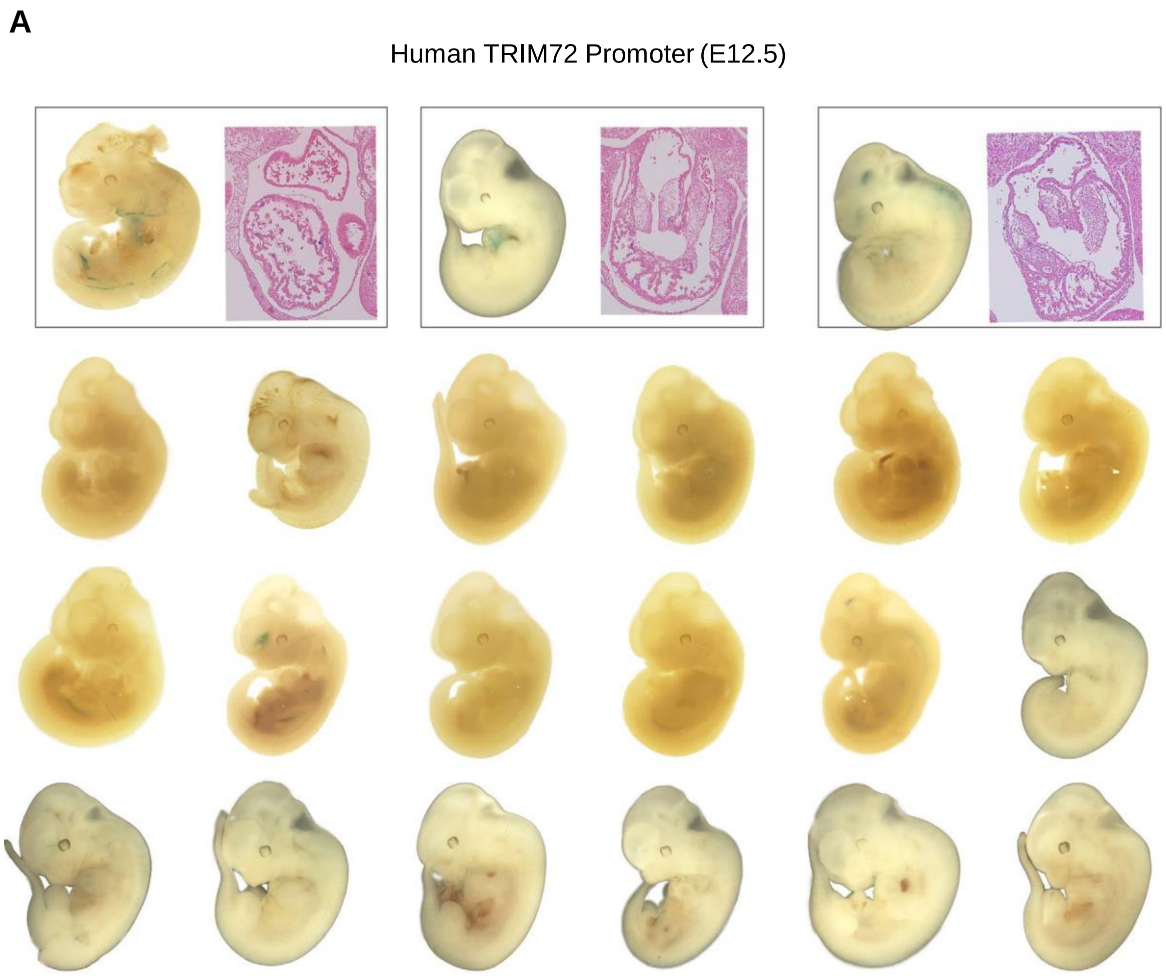

Figure S5

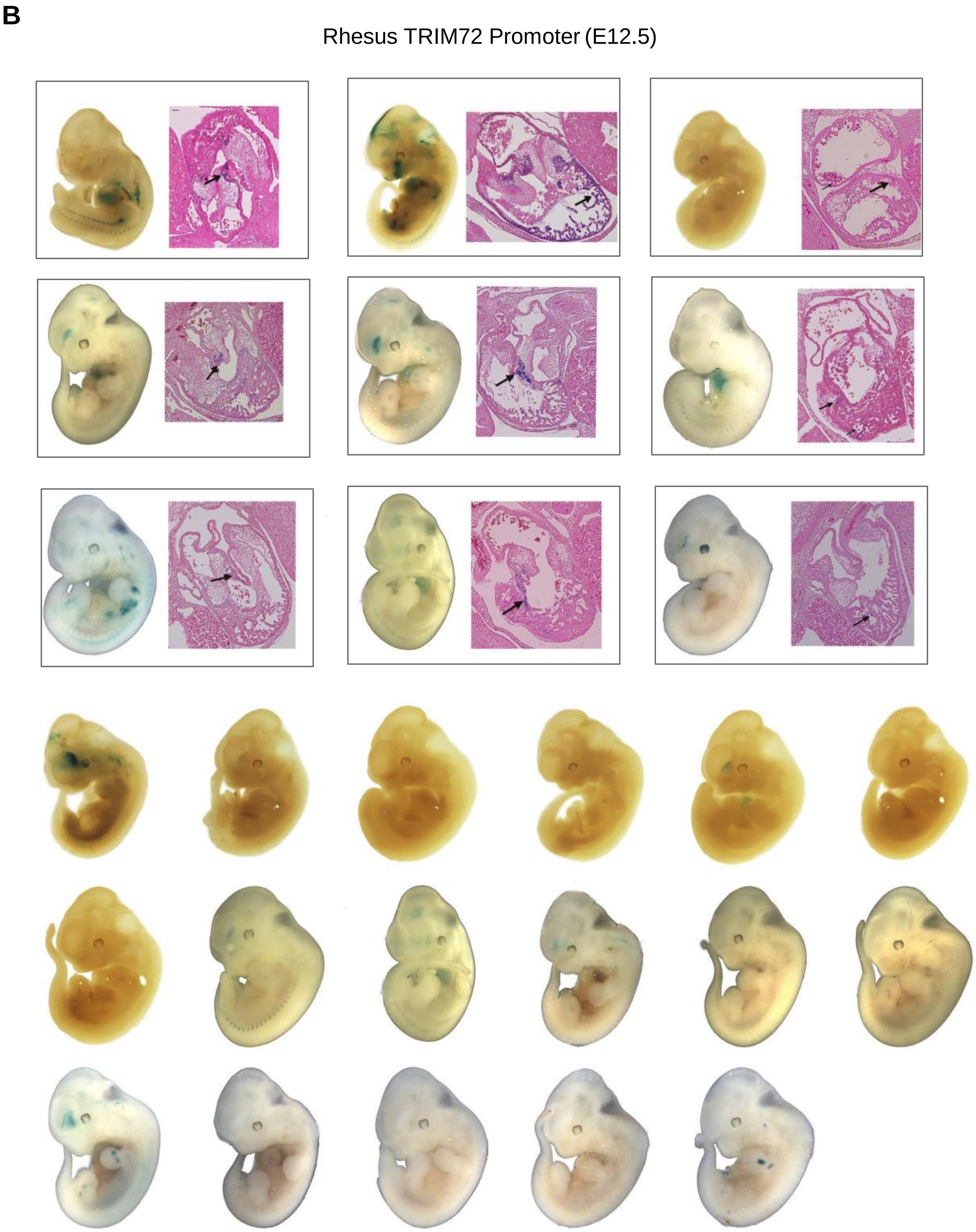

Figure S5. LacZ reporter transgenic assay of human and rhesus *TRIM72* promoter at E12.5 stage. Images of PCR-positive transgenic embryos at embryo day 12.5. A, human *TRIM72* promoter transgenic. B, rhesus *TRIM72* promoter transgenic. Embryos with positive LacZ in the heart were sliced and stained using eosin. LacZ positive sites are indicated with black arrows. Related to Figure 2B.

Figure S6

A

Human *TRIM72* Promoter (E13.5)

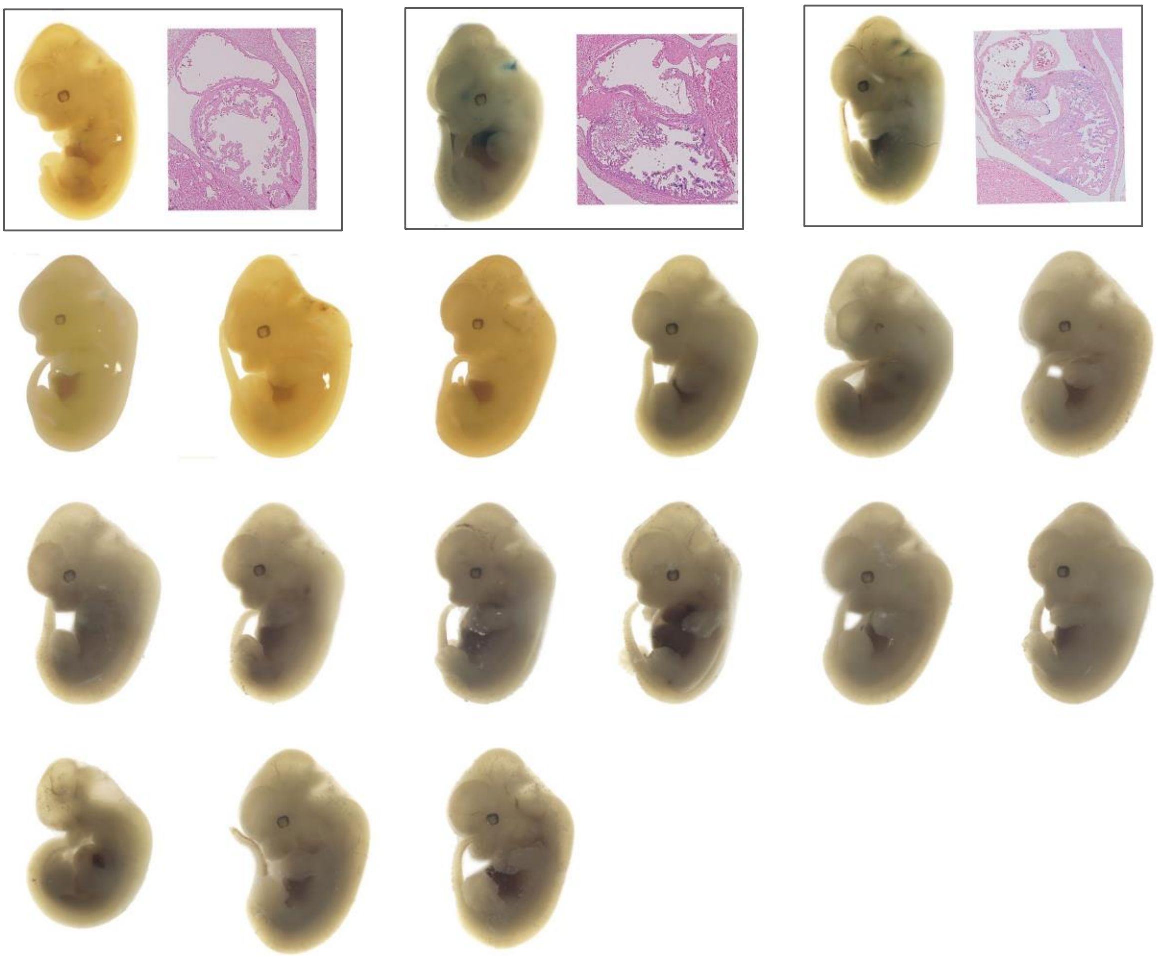

Figure S6

B

Rhesus *TRIM72* Promoter (E13.5)

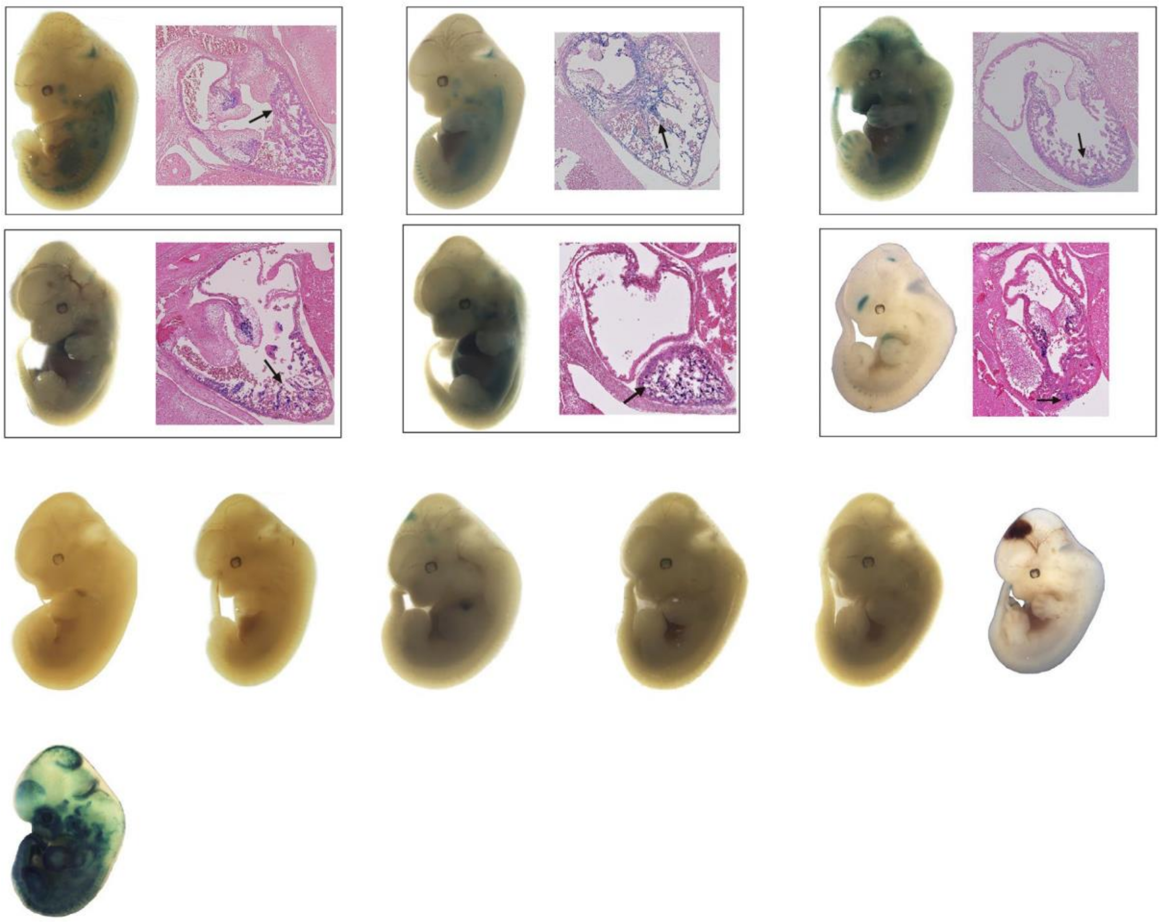

Figure S6. LacZ reporter transgenic assay of human and rhesus *TRIM72* promoters at E13.5 stage. Images of PCR-positive transgenic embryos at embryo day 13.5. A, human *TRIM72* promoter transgenic. B, rhesus *TRIM72* promoter transgenic. Embryos with positive LacZ in the heart were sliced and stained using eosin. LacZ positive sites are indicated with black arrows. Related to Figure 2C.

Figure S7

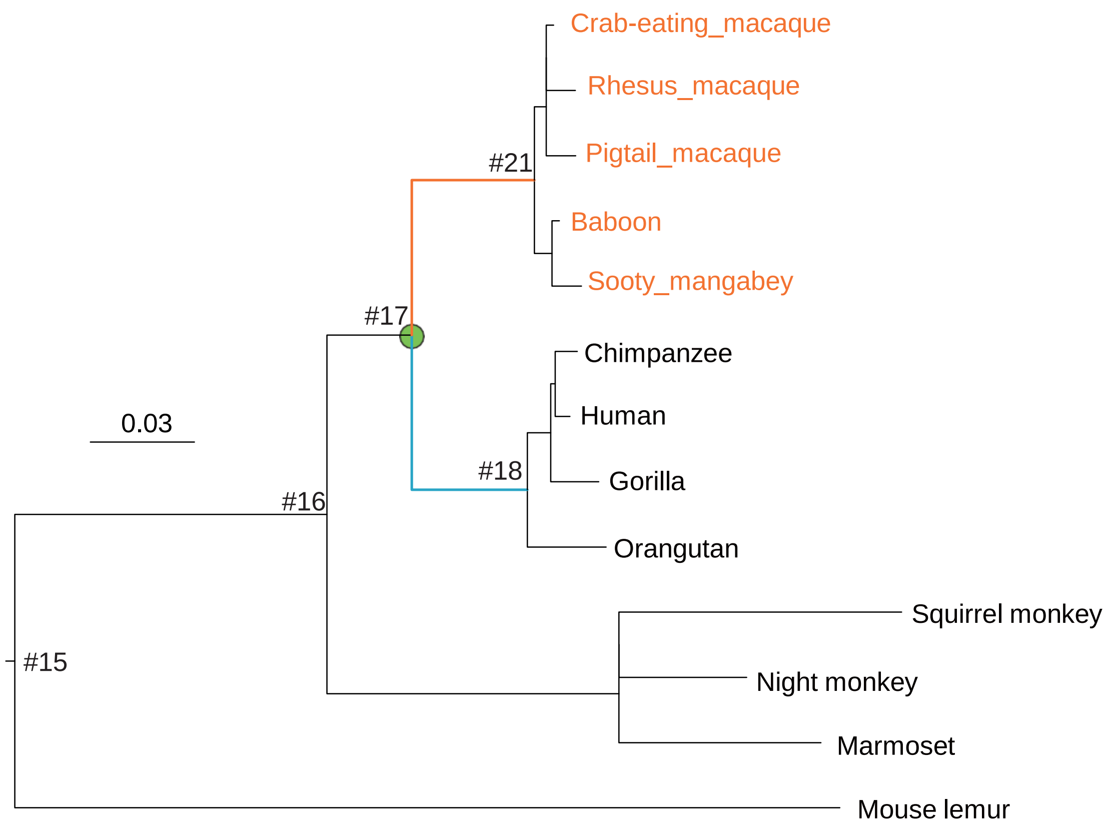

Figure S7. Maximum likelihood tree of 13 primates constructed by *TRIM72* CDS. The ancestral reconstruction of TRIM72 promoter sequences using the BASEML program under HKY8+Γ5 model in the PAML package. Numbers at the nodes indicate the common ancestors of Primates (#15), Simiiformes (#16), Catarrhini (#17), Hominoidea (#18), and Cercopithecidae (#21). Species with high TRIM72 expression in the heart are highlighted in orange. Related to Figure 3.

Figure S8

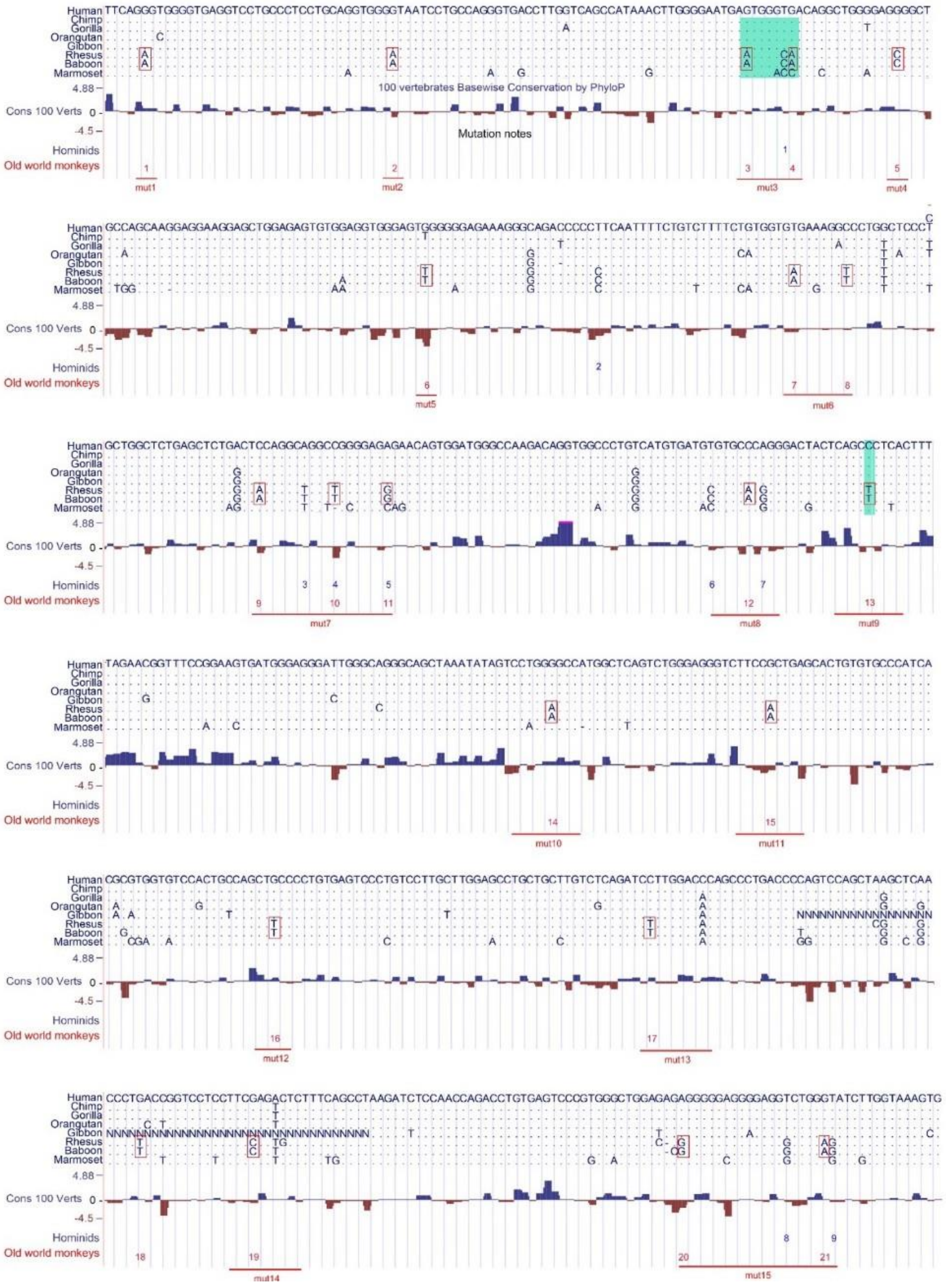

Figure S8. Sequence alignment of *TRIM72* promoters from 8 primate species. Red numbers indicate the rhesus-specific mutations compared with marmoset and human; these mutations are marked with red rectangles. Blue numbers indicate human-specific mutations compared with marmoset and rhesus. Red lines indicate the combined mutations used for promoter activity assay. The sequence alignment is from UCSC multiz alignments of 100 vertebrates (chr16: 31225015-31225675, hg19). Related to Figure 4.

Figure S9

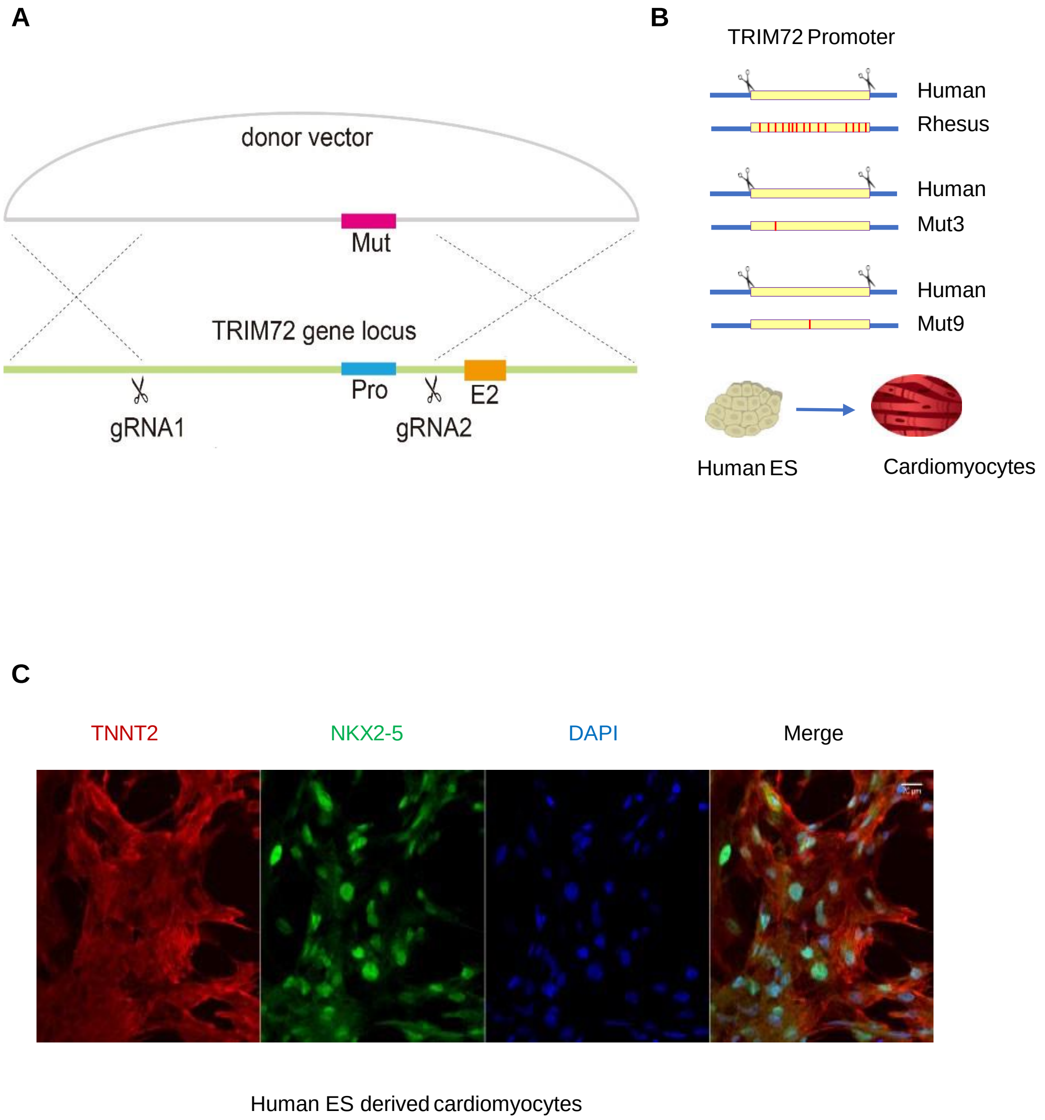

Figure S9. Characterization of cardiomyocytes derived from genetically edited hES cells. (A-B) Schematic diagram of genome editing of *TRIM72* promoter region in hES cells. Mut, mutated promoter. Pro, human *TRIM72* promoter. E2, human *TRIM72* exon 2. (C) Immunostaining of cardiomyocyte-specific markers (TNNT2 and NKX2-5) in hES-derived cardiomyocytes. Scale bar, 20 μm. Related to Figure 4D and 4E.

Figure S10

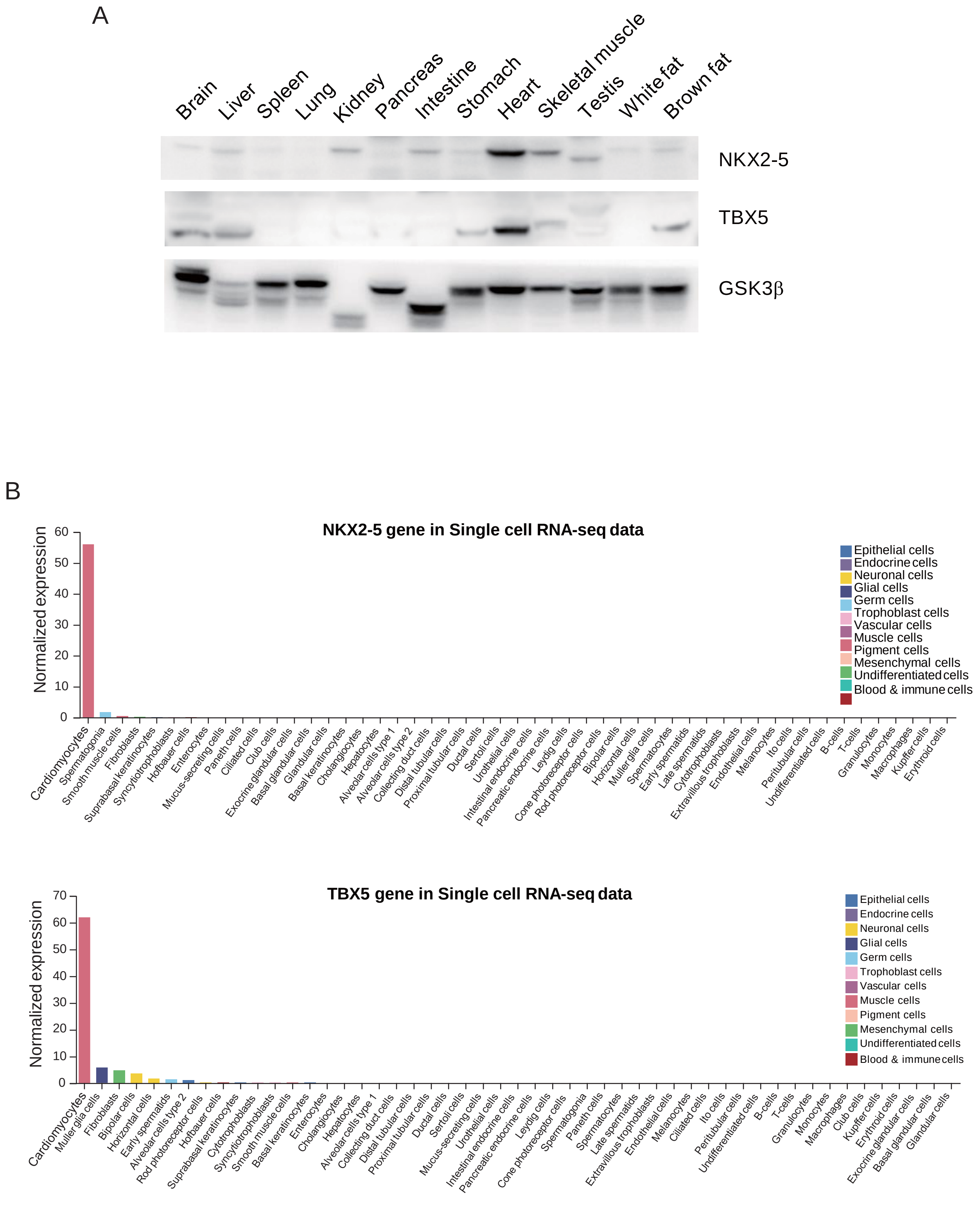

Figure S10. NKX2-5 and TBX5 are enriched in cardiomyocytes. (A) Western blots showing NKX2-5 and TBX5 are predominantly expressed in the murine heart. (B) RNA-seq of single cell type data showing NKX2-5 and TBX5 are specifically enriched in cardiomyocytes (data from https://www.proteinatlas.org/ENSG00000183072-NKX2-5/celltype and https://www.proteinatlas.org/ENSG00000089225-TBX5/celltype). Related to Figure 4F and 4G.

Figure S11

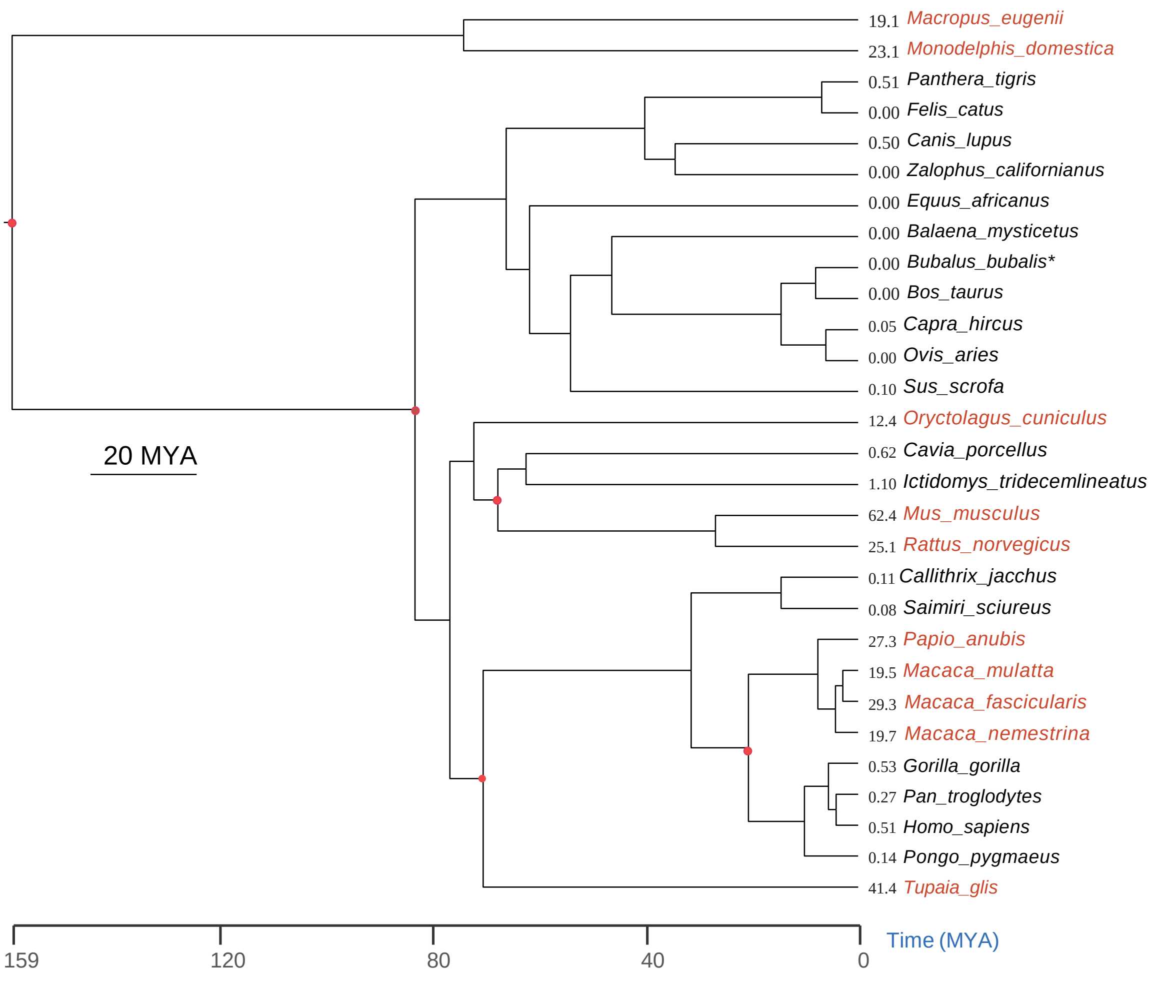

Figure S11. Maximum clade credibility tree of the 29 mammalian species. We created 10000 birth-death node-dated completed trees at http://vertlife.org/data/mammals and the trees are summarized by TreeAnnotator in BEAST v2.6.3. The MCC tree was visualized in Figtree v1.4.4 (http://tree.bio.ed.ac.uk/software/figtree/). The events in geological time and astronomical history are also downloaded from TIMETREE. The tree topology was manually double-checked to be consistent with published data. Red dots indicate the potential nodes where *cardiacTRIM72* expression changes happened.

Figure S12

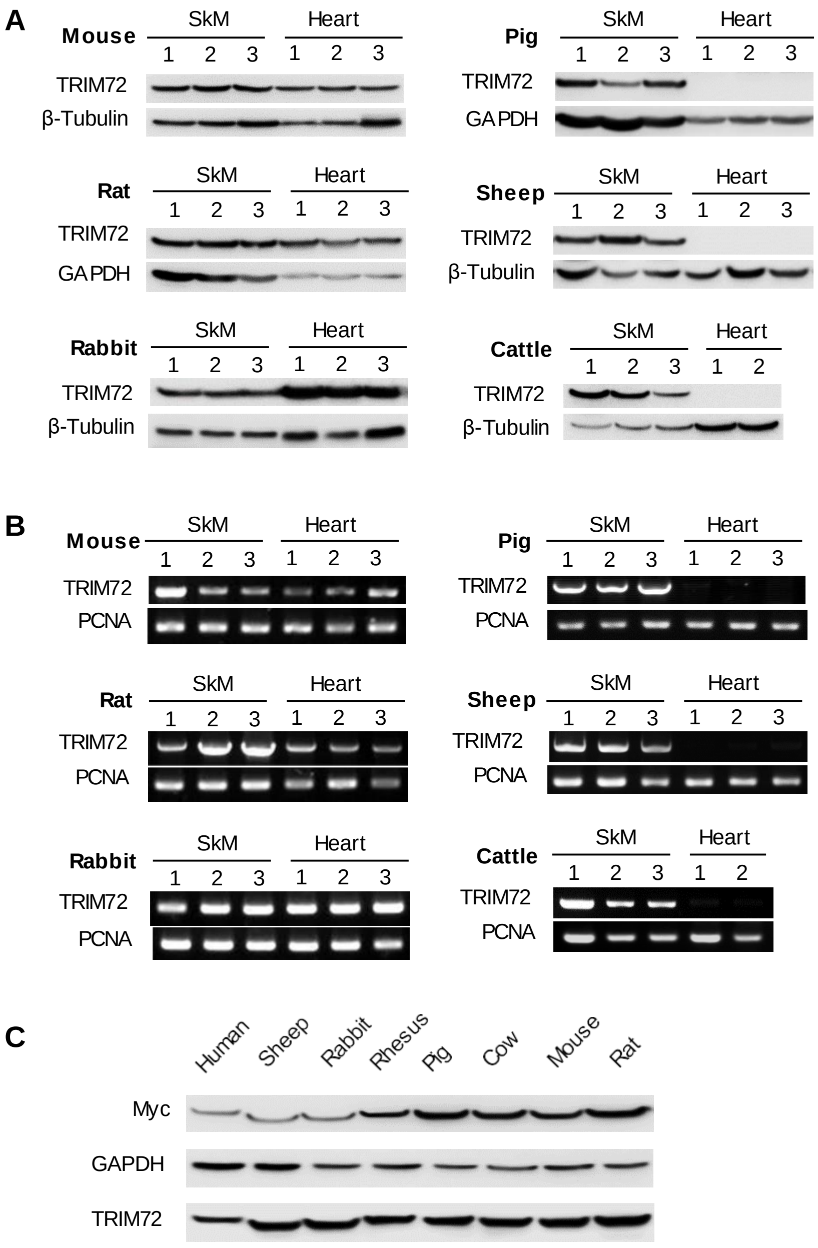

Figure S12. ***TRIM72*** **expression pattern in the heart of six mammalian species.** (A) Western blots showing the protein levels of *TRIM72* in the heart and skeletal muscle of 6 mammalian species. GAPDH or β-tubulin was used as loading control. (B) RT-PCR showing the mRNA levels of *TRIM72* in the heart and skeletal muscle of 6 mammalian species. PCNA was used as loading control. (C) The specificity of TRIM72 antibody was validated by Western blot across species. TRIM72 cDNAs from 8 species were cloned into an expression vector with Myc-tag and over-expressed in 293T cells, then the protein was identified by western blotting with either custom-made TRIM72 antibody or anti-Myc antibody.

Figure S13

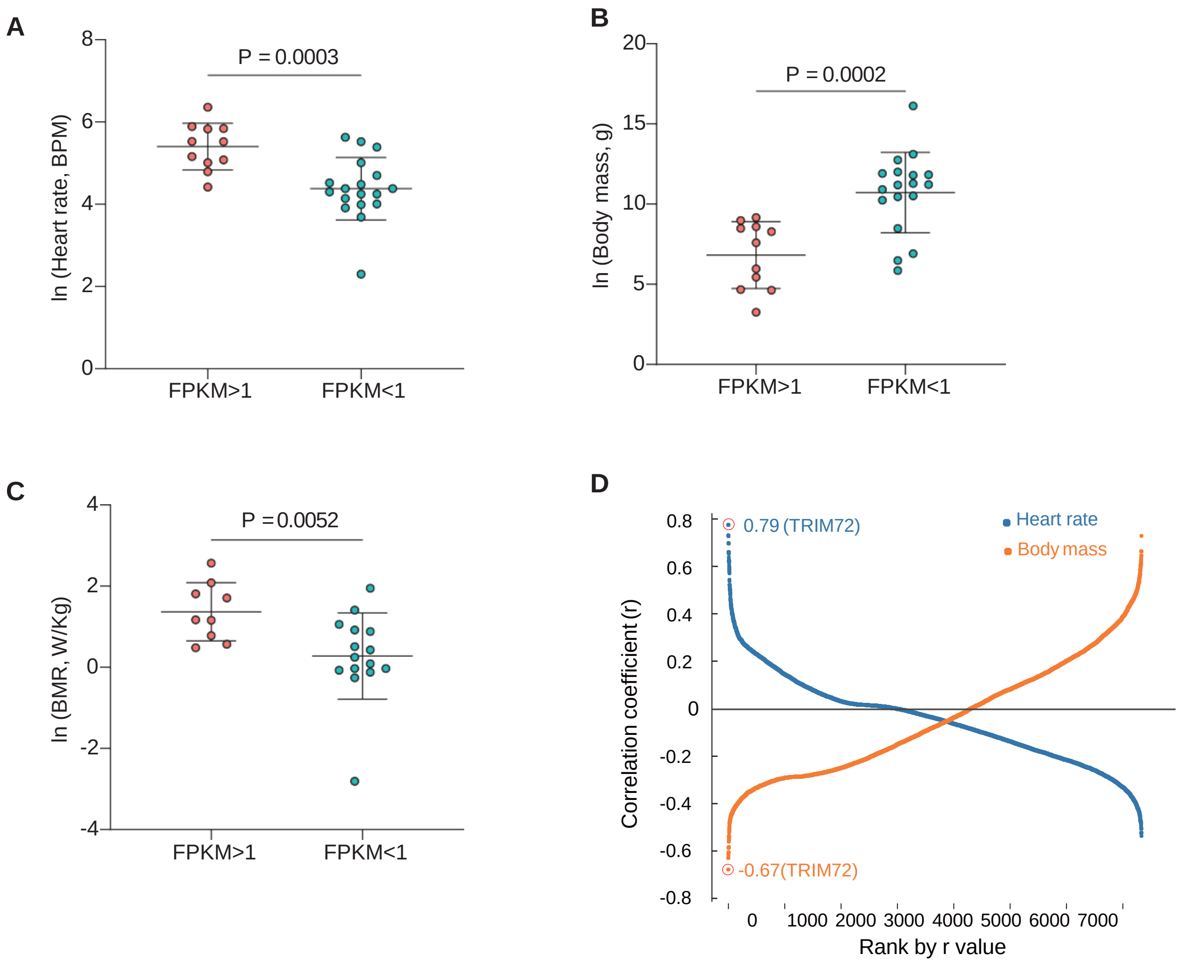

Figure S13. *TRIM72* expression is highly correlated with the heart rate and body mass in mammals. (A) Species with high cardiac TRIM72 have relatively higher heart rate (P = 0.0003). (B) Species with high cardiac TRIM72 have relatively lower body mass (P = 0.0002). (C) Species with high cardiac TRIM72 have relatively higher BMR per unit body weight (P = 0.0052). For statistics, species with high *TRIM72* expression (FPKM > 1) were compared with the low expression species (FPKM < 1) using Mann Whitney test. (D) Blue: Pearson correlation test between gene FPKM value and heart rate (R = 0.79 for *TRIM72*). Orange: Pearson correlation test between gene FPKM value and body mass (r = -0.67 for *TRIM72*). *TRIM72* is highlighted with red circles. 7335 gene from 20 representative species are used for the analysis.

Figure S14

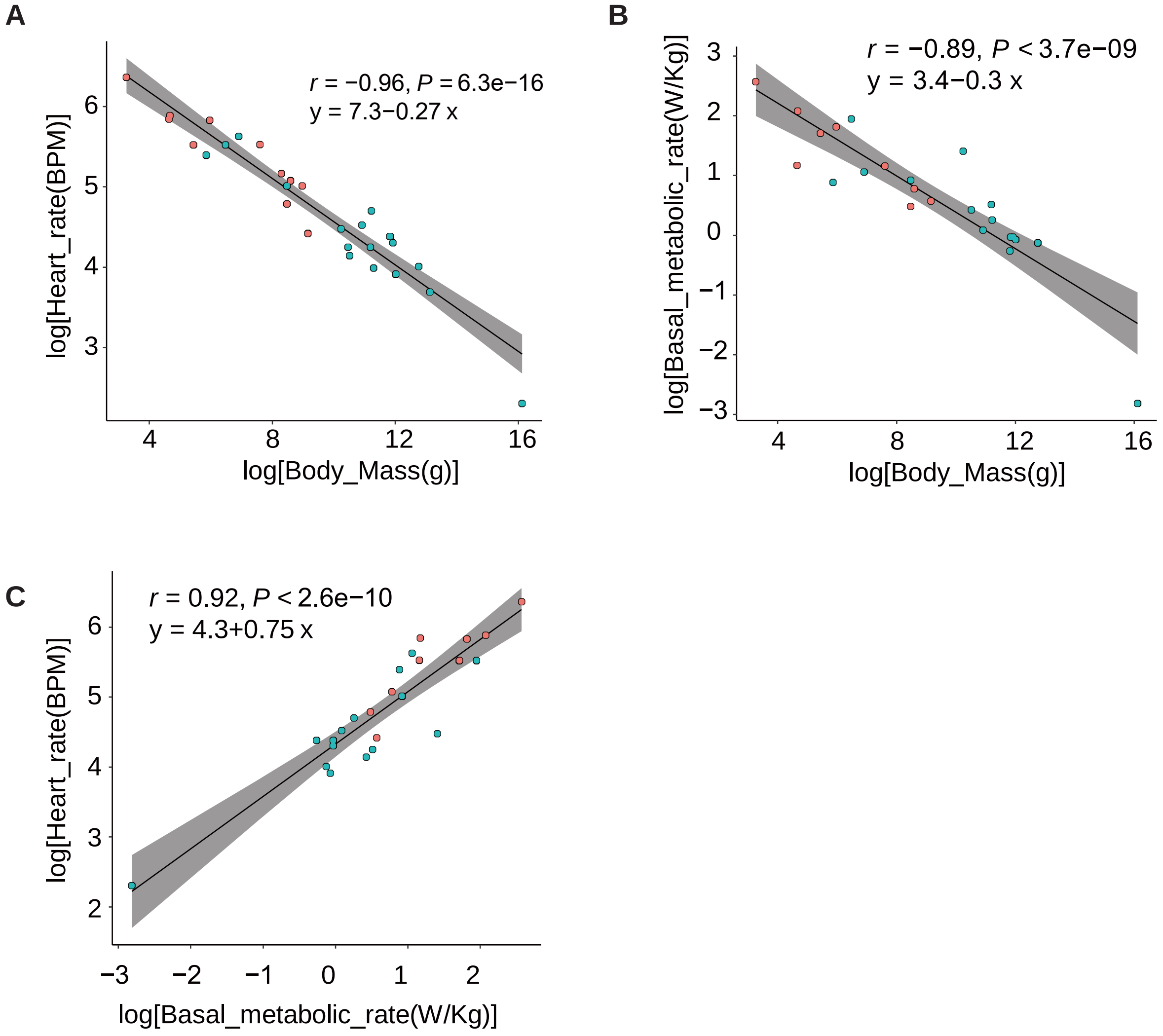

Figure S14. Allometric scaling between heart rate, body mass and BMR per unit body weight in 29 mammalian species. (A) Heart rate is negatively correlated with body mass. (B) BMR per unit body weight is negatively correlated with body mass. (C) Heart rate is positively correlated with BMR per unit body weight. Species are divided into two groups based on *TRIM72* expression levels in the heart. Species that highly express cardiac *TRIM72* (FPKM > 1) are marked in red.

Figure S15

A

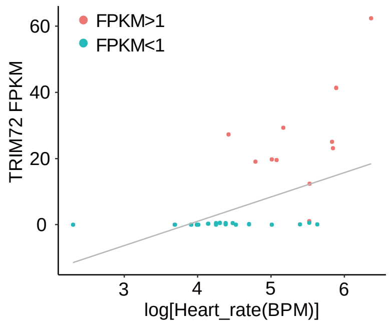

Coefficients	Estimate	StdErr	t.value	lowerbootCI	upperbootCI	p.value
(Intercept)	-28.4	10.7	-2.7	-51.9	-10.2	0.0130 *
log_HR	7.4	2.3	3.2	3.4	12.3	0.0035 **

R-squared: 0.2747 Adjusted R-squared: 0.2478

B

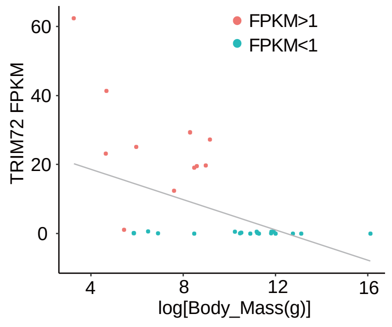

Coefficients	Estimate	StdErr	t.value	lowerbootCI	upperbootCI	p.value
(Intercept)	27.3	7.2	3.8	13.3	43.0	0.0008 ***
log_BM	-2.2	0.65	-3.4	-3.4	-0.89	0.0023 **

R-squared: 0.2946 Adjusted R-squared: 0.2685

C

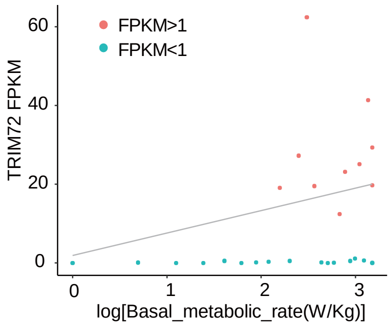

Coefficients	Estimate	StdErr	t.value	lowerbootCI	upperbootCI	p.value
(Intercept)	1.9	3.2	0.59	-5.6	8.3	0.56
log_HR	5.7	2.3	2.5	1.6	9.8	0.019 *

R-squared: 0.2256 Adjusted R-squared: 0.1904

D

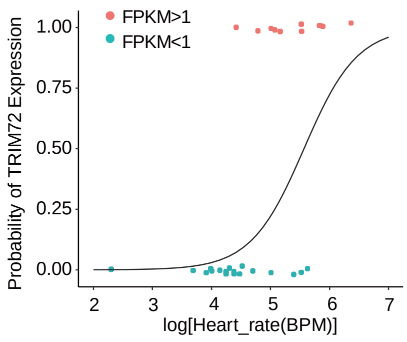

Coefficients	Estimate	StdErr	t.value	lowerbootCI	upperbootCI	p.value
(Intercept)	-12.4	5.3	-2.4	-15.3	-8.9	0.0172 *
log_HR	7.4	2.3	3.2	3.4	12.3	0.0035 **

E

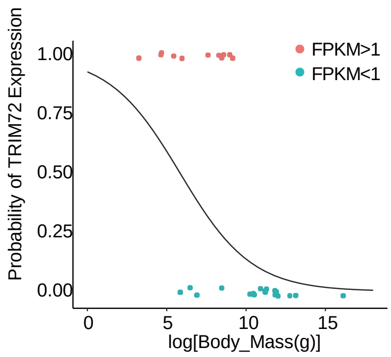

Coefficients	Estimate	StdErr	t.value	lowerbootCI	upperbootCI	p.value
(Intercept)	2.6	1.53	1.7	0.63	6.1	0.088
log_BM	-0.45	0.20	-2.2	-1.2	-0.15	0.028 *

F

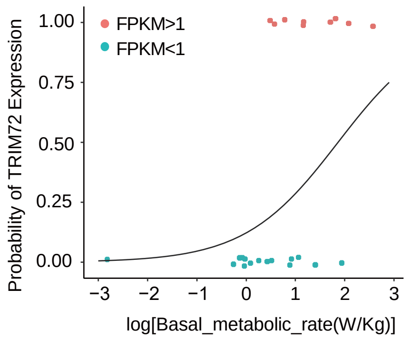

Coefficients	Estimate	StdErr	t.value	lowerbootCI	upperbootCI	p.value
(Intercept)	-1.9	1.4	-1.4	-3.3	0.32	0.160
log_BMR	1.1	0.61	1.7	0.06	2.2	0.083

Figure S15. Phylogenetic regressions show significant correlations between *TRIM72* expression and heart rate, body mass in mammals. (A) Phylogenetic linear regression of *TRIM72* FPKMs and log-transformed heart rate in 29 species. (B) Phylogenetic linear regression of *TRIM72* FPKMs and log-transformed body mass in 29 species. (C) Phylogenetic linear regression of *TRIM72* FPKMs and log-transformed Basal metabolic rate per unit weight in 24 species. (D) Phylogenetic logistic regression of *TRIM72* FPKMs and log-transformed heart rate in 29 species. (E) Phylogenetic logistic regression of *TRIM72* FPKMs and log-transformed body mass in 29 species. (F) Phylogenetic logistic regression of *TRIM72* FPKMs and log-transformed Basal metabolic rate per unit weight in 24 species. Blue dots indicate species with *TRIM72* cardiac FPKM < 1 and orange dots indicate species with *TRIM72* cardiac FPKM > 1. The coefficients and adjusted R-squared are shown for each regression analysis. Phylogenetic linear regression was performed under "OUrandomRoot" model, and logistic regression was performed using "logistic_IG10" method in Phylolm package with 100 bootstrap replicates.

Table S1. Transcription factor motif screening in human and rhesus *TRIM72* promoters. Related to Figure 3A, 4F and 4G.

TF binding analysis was performed online using JASPAR. Nine matrix profiles of heart-enriched transcription factors are used for motif scanning in human *TRIM72* promoter (chr16: 31224983-31225673, hg19) and rhesus *TRIM72* promoter (chr20: 28866360-28867045, rheMac8). These transcription factors included NKX2-5, TBX20, IRX4, TBX5, GATA4, IRX3, IRX5, GATA6 and HAND1. Transcription factor binding sites at mutation 3 (M3) and mutation 9 (M9) are highlighted in red.

Table S1.1 TF binding prediction in human *TRIM72* promoter

Matrix ID	Name	Score	Relative score	Start	End	Strand	Predicted sequence
MA0503.1	Nkx2-5(var.2)	3.224	0.805	4	14	+	CTCCCCTCAGC
MA0482.1	Gata4	4.290	0.833	21	31	-	TGTTTTCTTCC
MA1104.1	GATA6	5.880	0.807	21	33	+	GGAAGAAAACATT
MA0807.1	TBX5	5.302	0.805	37	44	+	GGGTGGGG
MA0807.1	TBX5	6.455	0.835	40	47	+	TGGGGTGA
MA0807.1	TBX5	7.410	0.859	64	71	+	AGGTGGGG
MA0503.1	Nkx2-5(var.2)	7.644	0.871	113	123	-	ACCCACTCATT
MA0807.1	TBX5	5.833	0.819	120	127	+	GGGTGACA
MA0503.1	Nkx2-5(var.2)	7.228	0.864	162	172	-	ACACTCTCCAG
MA0807.1	TBX5	5.515	0.810	167	174	+	GAGTGTGG
MA0807.1	TBX5	9.764	0.920	175	182	+	AGGTGGGA
MA0482.1	Gata4	3.121	0.817	188	198	-	CCCTTTCTCCC
MA0092.1	Hand1::Tcf3	6.489	0.815	195	204	-	GGTCTGCCCT
MA0092.1	Hand1::Tcf3	6.619	0.819	214	223	+	TTTCTGTCTT
MA1104.1	GATA6	9.201	0.868	218	230	-	CACAGAAAAGACA
MA0482.1	Gata4	7.313	0.877	220	230	+	TCTTTTCTGTG
MA0689.1	TBX20	11.722	0.892	228	238	+	GTGGTGTGAAA
MA0807.1	TBX5	9.804	0.921	229	236	+	TGGTGTGA
MA0092.1	Hand1::Tcf3	6.846	0.825	270	279	-	TGCCTGGAGT
MA0482.1	Gata4	7.396	0.878	285	295	-	TGTTCTCTCCC
MA1104.1	GATA6	5.499	0.800	285	297	+	GGGAGAGAACAGT
MA0503.1	Nkx2-5(var.2)	3.390	0.808	329	339	-	GGGCACACATC
MA0503.1	Nkx2-5(var.2)	4.913	0.830	343	353	+	GACTACTCAGC
MA0092.1	Hand1::Tcf3	7.283	0.838	433	442	+	AGTCTGGGAG
MA0807.1	TBX5	6.023	0.823	470	477	-	ACGCGTGA
MA0092.1	Hand1::Tcf3	6.838	0.825	486	495	-	CAGCTGGCAG
MA0482.1	Gata4	5.221	0.847	531	541	+	GCTTGTCTCAG
MA0482.1	Gata4	2.153	0.803	568	578	-	GCTTAGCTGGA
MA0092.1	Hand1::Tcf3	9.192	0.894	626	635	-	GGTCTGGTTG
MA0482.1	Gata4	3.874	0.827	652	662	-	CCCTCTCTCCA
MA0092.1	Hand1::Tcf3	8.004	0.859	671	680	+	GGTCTGGGTA

Table S1.2 TF binding prediction in rhesus *TRIM72* promoter

Matrix ID	Name	Score	Relative score	Start	End	Strand	Predicted sequence
MA0503.1	Nkx2-5(var.2)	3.224	0.805	4	14	+	CTCCCCTCAGC
MA0482.1	Gata4	4.290	0.833	21	31	-	TGTTTTCTTCC
MA1104.1	GATA6	5.880	0.807	21	33	+	GGAAGAAAACATT
MA0503.1	Nkx2-5(var.2)	3.726	0.813	34	44	-	CCCCACTCTGA
MA0807.1	TBX5	6.455	0.835	40	47	+	TGGGGTGA
MA0807.1	TBX5	9.764	0.920	64	71	+	AGGTGGGA
MA0503.1	Nkx2-5(var.2)	7.228	0.864	162	172	-	ACACTCTCCAG
MA0807.1	TBX5	5.515	0.810	167	174	+	GAGTGTGG
MA0807.1	TBX5	9.764	0.920	175	182	+	AGGTGGGA
MA0482.1	Gata4	3.121	0.817	188	198	-	CCCTTTCTCCC
MA0807.1	TBX5	6.124	0.826	195	202	+	AGGGGAGA
MA0807.1	TBX5	6.415	0.834	203	210	-	AGGGGGGA
MA0092.1	Hand1::Tcf3	6.619	0.819	215	224	+	TTTCTGTCTT
MA1104.1	GATA6	9.201	0.868	219	231	-	CACAGAAAAGACA
MA0482.1	Gata4	7.313	0.877	221	231	+	TCTTTTCTGTG
MA0807.1	TBX5	6.422	0.834	230	237	+	TGGTGAGA
MA0482.1	Gata4	3.683	0.825	230	240	-	CTTTCTCACCA
MA0482.1	Gata4	3.466	0.822	242	252	+	TCCTGTCTCCC
MA0503.1	Nkx2-5(var.2)	4.018	0.817	263	273	+	GAGCTCTGAGG
MA0807.1	TBX5	5.302	0.805	276	283	+	GGGTGGGG
MA0092.1	Hand1::Tcf3	6.253	0.808	313	322	+	GCCCTGGCAT
MA0503.1	Nkx2-5(var.2)	4.913	0.830	339	349	+	GACTACTCAGC
MA0807.1	TBX5	6.596	0.838	350	357	-	AAGTGAGA
MA0092.1	Hand1::Tcf3	7.283	0.838	429	438	+	AGTCTGGGAG
MA0807.1	TBX5	6.023	0.823	466	473	-	ACGCGTGA
MA0092.1	Hand1::Tcf3	6.032	0.801	482	491	-	AAGCTGGCAG
MA0482.1	Gata4	5.221	0.847	527	537	+	GCTTGTCTCAG
MA0092.1	Hand1::Tcf3	9.192	0.894	622	631	-	GGTCTGGTTG
MA0092.1	Hand1::Tcf3	7.076	0.832	644	653	+	GGGCTGGAAG
MA0503.1	Nkx2-5(var.2)	3.865	0.815	669	679	-	AAGATCTCCAG

Table S2. The physiological parameters and *TRIM72* FPKM values of 29 mammalian species.

All mammalian physiological parameters including body mass, heart rate (HR) and basal metabolic rate (BMR) were collected from public databases or papers. The unit of BMR was converted to Watt/Kg using following formula: 1W = 0.0143 kcal/min = 1 Joule/second and 1 L of O_2 = 5.05 kcal. References for the physiological parameters are available upon request.

Names	Species	FPKM	Heart Rate (BPM)	Body_Mass(g)	BMR(W/kg)	RNA Data Source
Mouse	*Mus musculus*	62.42	580	26	13.08	SRR207096
Tree shrew	*Tupaia chinensis*	41.36	360	107	7.97	GSE39150
Baboon	*Papio anubis*	27.25	83	9500	1.77	NHPRTR
Rat	*Rattus norvegicus*	25.09	340	390	6.14	SRR594439
Opposusm	*Monodelphis domestica*	23.14	345	104	3.22	SRP023152
Rhesus Macaque	*Macaca mulatta*	19.53	160	5380	2.18	NHPRTR
Wallaby	*Macropus eugenii*	19.09	120	4796	1.62	DRX012250
Rabbit	*Oryctolagus cuniculus*	12.38	251	2000	3.18	SRR1789341
Pigtail Macaque	*Macaca nemestrina*	19.71	150	7913	N/A	NHPRTR
Crab-eating Macaque	*Macaca fascicularis*	29.33	175	4000	N/A	NHPRTR
Gorilla	*Gorilla gorilla*	0.53	74	150000	0.97	SRR306805
Human	*Homo sapiens*	0.51	70	72200	1.67	NHPRTR
Tiger	*Panthera tigris altaica*	0.51	80	137900	0.97	SRR1015468
Dog	*Canis lupus familiaris*	0.50	88	28000	4.08	SRP009687
Chimpanzee	*Pan troglodytes*	0.27	63	36900	1.53	NHPRTR
Orangutan	*Pongo pygmaeus*	0.14	110	74800	1.29	SRR306795
Marmoset	*Callithrix jacchus*	0.11	220	350	2.42	NHPRTR
Pig	*Sus scrofa*	0.10	80	135000	0.77	SRR3160018
Squirrel Monkey	*Saimiri sciureus*	0.08	278	1000	2.88	NHPRTR
Goat	*Capra hircus*	0.05	70	35000	N/A	GSE37456
Cat	*Felis catus*	0.00	150	4800	2.51	SRP071078
Bowhead whale	*Balaena mysticetus*	0.00	10	10000000	0.06	SRX790346
Aisa buffalo	*Bubalus bubalis*	0.00	40	500000	N/A	ERP003627
Cattle	*Bos taurus*	0.00	55	347000	0.88	SRR594484
Sheep	*Ovis aries*	0.00	92	55000	1.09	SRR1230347
Ground Squirrel	*Ictidomys tridecemlineatus*	1.10	250	230	5.53	SRR087419.2
Domestic guinea pig	*Cavia porcellus*	0.62	250	650	7.00	SRR6411088
California_sea_lion	*Zalophus californianus*	0.00	54	80000	N/A	SRR1981987
Guanzhong_donkey	Equus africanus	0.00	50	164900	0.93	SRR8184587

Table S3. Primers used for luciferase plasmid construction in promoter truncation assay in Figure 2A.

Restriction enzyme sites are marked in red.

Fragments	PGL4 Primer	Sequence (5' to 3')
Hu-690bp	PGL4-53P-kpn1-u	ATATGGTACCCTCCCCTCAGCTCTGGAGGAAG
	PGL4-hu-53P-hind3-d	CTCTAAGCTTTACCAAGATACCCAGACCTCC
Rh-690bp	PGL4-53P-kpn1-u	ATATGGTACCCTCCCCTCAGCTCTGGAGGAAG
	PGL4-Rh-53P-hind3-d	CTCTAAGCTTTACCAAGATCTCCAGCCCTCC
Rh-600bp	PGL4-53P-hind3-d1	ATGAAGCTTGTAAGGGTCGAGCCGAGCTGGAC
Rh-490bp	PGL4-53P-kpn1-u1	GCCGGTACCCCTCAATTTTCTGTCTTTTCTG
Rh-400bp	PGL4-53P-kpn1-u2	ATAGGTACCTGGATGGGCCAAGACAGGTGGC
Rh-330bp	PGL4-53P-kpn1-u3	ATAGGTACCTAGAACGGTTTCCGGAAGTGATGGG
Rh-230bp	PGL4-53P-kpn1-u4	ATAGGTACCCATCACGCGTGGTGTCCACTGCCAG

Table S4. Primers used for rhesus *TRIM72* promoter mutation assay in Figure 4A.

Mutation	Name	Sequence (5'to3')
mut1	rh2hu-mut1-u	CACCCCACCCTGAAATGTTTTCTTCCTCCAGAGCTG
	rh2hu-mut1-d	AACATTTCAGGGTGGGGTGAGGTCCTGCCCTCCTGC
mut2	rh2hu-mut2-u	TGGCAGGATTACCCCACCTGCAGGAGGGCAGGACCT
	rh2hu-mut2-d	AGGTGGGGTAATCCTGCCAGGGTGACCTTGGTCAG
mut3	rh2hu-mut3-u	GCCTGTCACCCACTCATTCCCCAAGTTTATGGCTGACC
	rh2hu-mut3-d	GGAATGAGTGGGTGACAGGCTGGGGAGCGGCTGCCAGCAA
mut4	rh2hu-mut4-u	GCTGGCAGCCCCTCCCCAGCCTGTTGCCCATTCATT
	rh2hu-mut4-d	AGGCTGGGGAGGGGCTGCCAGCAAGGAGGAAGGAGCT
mut5	rh2hu-mut5-u	CTCCCCCCACTCCCACCTCCACACTCTCCAGCTCC
	rh2hu-mut5-d	GTGGGAGTGGGGGGAGAAAGGGGAGATCCCCCCTCA
mut6	rh2hu-mut6-u	CCAGGGCCTTTCACACCACAGAAAAGACAGAA
	rh2hu-mut6-d	TTCTGTGGTGTGAAAGGCCCTGGCTCCCCGCT
mut7	rh2hu-mut7-u	TCTCCCCGGCCTGCCTGGAGTCAGAGCTCAGAGCCA
	rh2hu-mut7-d	TGACTCCAGGCAGGCCGGGGAGAGAACAGTGGAT
mut8	rh2hu-mut8-u	CCCTGGGCACACATCACATGCCAGGGCCACCTGTCTTGG
	rh2hu-mut8-d	GTGATGTGTGCCCAGGGACTACTCAGCTCTCACTTTTAG
mut9	rh2hu-mut9-u	TACTCAGCCCTCACTTTTAGAACGGTTTCCGGAAG
	rh2hu-mut9-d	AAAGTGAGGGCTGAGTAGTCCCCGTGCACGCATCACAT
mut10	rh2hu-mut10-u	GCGGAAGACCCTCCCAGACTGAGCCATGGCCCCAGGAC
	rh2hu-mut10-d	GGGCCATGGCTCAGTCTGGGAGGGTCTTCCGCTGAGCAC
mut11	rh2hu-mut11-u	TGCCAGCTGCCCCTGTGAGTCCCTGTCCTTGCTTGG
	rh2hu-mut11-d	TCACAGGGGCAGCTGGCAGTGGACACCACGCGTGAT
mut12	rh2hu-mut12-u	GGCTGGGTCCAAGGATCTGAGACAAGCAGCAGGC
	rh2hu-mut12-d	TCTCAGATCCTTGGACCCAGCCCTGACCCCAGTC
mut13	rh2hu-mut13-u	GACCGGTCCTCCTTCGAGACTCTTTCAGCCTAAGATCTCCAA
	rh2hu-mut13-d	GTCTCGAAGGAGGACCGGTCAGGGTCGAGCCTAGCTGGACTG
mut14	rh2hu-mut14-u	GCTAAGCTCAACCCTTACCGGTCCTCCTTCCAGTGTCTTTCAGCCTA
	rh2hu-mut14-d	AGGGTTGAGCTTAGCTGGACTGGGGTCAGGGCTG
mut15	rh2hu-mut15-u	GATACCCAGACCTCCCCTCCCCCTCTCTCCAGCCCACG
	rh2hu-mut15-d	GAGAGAGGGGGAGGGGAGGTCTGGGTATCTTGGTAAAG

Table S5. Primers used for human *TRIM72* promoter mutation assay in Figure 4B.

Mutation	Name	Sequence (5' to 3')
Mut1	hu2rh-mut1-u	CAGGACCTCACCCCACTCTGAAATGTTTTCTTC
	hu2rh-mut1-d	GAAGAAAACATTTCAGAGTGGGGTGAGGTCCTG
Mut2	hu2rh-mut2-u	CCTGGCAGGATTATCCCACCTGCAGGAG
	hu2rh-mut2-d	CTCCTGCAGGTGGGATAATCCTGCCAGG
Mut3	hu2rh-mut3-u	CCCAGCCTGTTGCCCATTCATTCCCCAAGTTTATG
	hu2rh-mut3-d	CATAAACTTGGGGAATGAATGGGCAACAGGCTGGG
Mut4	hu2rh-mut4-u	TCCTTGCTGGCAGCCGCTCCCCAGCCT
	hu2rh-mut4-d	AGGCTGGGGAGcGGCTGCCAGCAAGGA
Mut5	hu2rh-mut5-u	CCTTTCTCCCCCAACTCCCACCTCCACAC
	hu2rh-mut5-d	GTGTGGAGGTGGGAGTTGGGGGAGAAAGG
Mut6	hu2rh-mut6-u	GAGCCAGGACCTTTCTCACCACAGAAAAGAC
	hu2rh-mut6-d	GTCTTTTCTGTGGTGAGAAAGGTCCTGGCTC
Mut7	hu2rh-mut7-u	ACTGTTCCCTCCCCAGCCAGCCTGTAGCCAGAGCTCAGAG
	hu2rh-mut7-d	CTCTGAGCTCTGGCTACAGGCTGGCTGGGGAGGGAACAGT
Mut8	hu2rh-mut8-u	GGGCTGAGTAGTCCCCGTGCACGCATCACATGAC
	hu2rh-mut8-d	GTCATGTGATGCGTGCACGGGGACTACTCAGCCC
Mut9	hu2rh-mut9-u	GTTCTAAAAGTGAGAGCTGAGTAGTCCCTG
	hu2rh-mut9-d	CAGGGACTACTCAGCTCTCACTTTTAGAAC
Mut10	hu2rh-mut10-u	CAGACTGAGCCATGGCTCCAGGACTATATTTAG
	hu2rh-mut10-d	CTAAATATAGTCCTGGAGCCATGGCTCAGTCTG
Mut11	hu2rh-mut11-u	CACACAGTGCTCAGTGGAAGACCCTCCCAG
	hu2rh-mut11-d	CTGGGAGGGTCTTCCACTGAGCACTGTGTG
Mut12	hu2rh-mut12-u	GACTCACAGGGGAAGCTGGCAGTGGACAC
	hu2rh-mut12-d	GTGTCCACTGCCAGCTTCCCCTGTGAGTC
Mut13	hu2rh-mut13-u	GGTCAGGGCTGTGTCCAAAGATCTGAGACAAGCAGC
	hu2rh-mut13-d	GCTGCTTGTCTCAGATCTTTGGACACAGCCCTGACC
Mut14	hu2rh-mut14-u	ATCTTAGGCTGAAAGACACTGGAAGGAGGACCGGTCAG
	hu2rh-mut14-d	GACCGGTCCTCCTTCCAGTGTCTTTCAGCCTAAGATCTC
Mut15	hu2rh-mut15-u	GCTTTACCAAGATCTCCAGCCCTCCCCTCCCCCTCTCTC
	hu2rh-mut15-d	GGAGGGGAGGGCTGGAGATCTTGGTAAAGCTTGGC

Table S6. RT-qPCR primers used for Figure 6A and 6B.

RT-qPCR Primer	Sequence (5' to 3')
Rat-Sdha-u	GACGGGCCACTCACTCTTAC
Rat-Sdha-d	AAGGTAAACCAGCCCGAGTG
Rat-Ndufa5-u	TTTGCGACACTCCACACGAG
Rat-Ndufa5-d	GGCCACTTCCACTGGTTAGC
Rat-Ndufv3-u	ATAAAAAGGCGGCAGGACCAA
Rat-Ndufv3-d	TAAGAACTGGGTGGAGGGGAG
Rat-Cox6a1-u	GAAGGCCCTCACCTACTTCG
Rat-Cox6a1-d	ATCCAATGGGCTTTCGTGGT
Rat-Uqcrc1-u	GCACTACAAAGCTCCCCGAA
Rat-uqcrc1-d	AGCAACCCGGTCTCAGAGTA
Rat-Uqcrfs1-u	GGTTCTGGACGTGAAGCGA
Rat-Uqcrfs1-d	CTAGAACTTCAGCACGGCGA
Rat-Actb-u	TGTACCCAGGCATTGCTGAC
Rat-Actb-d	AAAACGCAGCTCAGTAACAGTC
GAPDH-u	GTGGACCTGACCTGCCGTCT
GAPDH-d	GGAGGAGTGGGTGTCGCTGT
hTRIM72-u	ACCAGCAGGCTGCAGAAGAT
hTRIM72-d	TCCTCCACACCTGGAATTTGA
18S-u	GGAAGGGCACCACCAGGAGT
18S-d	TGCAGCCCCGGACATCTAAG

Table S7. Primers used for *TRIM72* ChIP-qPCR in NRVMs in Figure 6C.

ChIP-qPCR Primer	Sequence (5' to 3')
Ndufa5-rat-ChIP-u	GAGGGAGAGATTTGCGCCT
Ndufa5-rat-ChIP-d	TTTTCGTAGCGCAGCGTTTT
Ndufv3-rat-ChIP-u	TCTCACCCGGGATTAGTGGA
Ndufv3-rat-ChIP-d	TCACGTTCGCCAATCGTACA
Sdha-rat-ChIP-u	CTCACAGGTAATGCCACCGC
Sdha-rat-ChIP-d	TCAGCGATAGACTGCGCTTG
Cox6a1-rat-ChIP-u	AAGGCGTTGCCCAATGAAAC
Cox6a1-rat-ChIP-d	CAGAGACTCGAGACGCACTC
Uqcrfs1-rat-ChIP-u	GTGAAGCGACCCTTCCTGTG
Uqcrfs1-rat-ChIP-d	CAGGGAAACGAGATCGGGG
Uqcrc1-rat-ChIP-u	GACCAAACGACAACATGCGA
Uqcrc1-rat-ChIP-d	AACTTCCGTCAGCGTAAAGC

Table S8. RT-PCR Primers used for TRIM72 expression test in Figure S12B.

RT-PCR Primer	Sequence (5' to 3')
Rat-RT-u	GCCACCATGTCGACTGCACCAGGCCTTTTGC
Rat-RT-d	GGCCTGCTCACTGTCTGGCCCCACGA
Mouse-RT-u	ACGCCTCAAGACACAGCTTCCACAGC
Mouse-RT-d	GAGCACTATTGGGAGGTGGAG
Pig/rabbit/sheep/cow-RT-u	CCGCTGTGCCTGCAGCTGTTC
Pig/rabbit/sheep/cow-RT-d	CTCCACCTCCCAGTAGTGCTC
RT-PCNA-u	TGAACCTCACCAGCATGTCCAA
RT-PCNA-d	CGGCATATACGTGCAAATTCAC

Table S9. Primers used for cloning of *TRIM72* coding sequences in Figure S12C.

Peasy-M2 Primer	Sequence (5' to 3')
Rabbit-TRIM72-CDS-u	GCCACCATGTCGGCCGCGCCCGGCCTCCTGCA
Rabbit-TRIM72-CDS-d	GGCCTCCGGGCATCCGGCCCCACGA
Sheep-TRIM72-CDS-u	GCCACCATGTCGGCCGCGCCCGGCCTCCTGCA
Sheep-TRIM72-CDS-d	GGCCTCCCCGCCGCTGTCGGGCCCCA
Pig-TRIM72-CDS-u	GCCACCATGTCAGCTGCGCCCGGCCTCCTGC
Pig-TRIM72-CDS-d	GGCCTCCCCGCCGCTGTCGGGCCCCA
Cow-TRIM72-CDS-u	GCCACCATGTCGGCCGCGCCCGGCCTCCTGCA
Cow-TRIM72-CDS-d	AGCCTCCGAGCCGGAGCCGCTGCTG
Rat-TRIM72-CDS-u	GCCACCATGTCGACTGCACCAGGCCTTTTGC
Rat-TRIM72-CDS-d	GGCCTGCTCACTGTCTGGCCCCACGA
Rhesus-TRIM72-CDS-u	GCCACCATGTCGGCTGCGCCGGGCCTCCT
Rhesus-TRIM72-CDS-d	GGCTTCGGCGCCTTCGGACCCCAC
Mouse-TRIM72-CDS-u	GCCACCATGTCGGCTGCACCCGGCCTTCTG
Mouse-TRIM72-CDS-d	GGCCTGTTCCTGCTCCGGCCCCAC
Human-TRIM72-CDS-u	GCCACCATGATGTCGGCTGCGCCCGGCCTCCTG
Human-TRIM72-CDS-d	GGCCTCGGCGCCTTCGGGACCCAC
